# Pharmacological Inhibition of PP2A Overcomes Nab-Paclitaxel Resistance by Downregulating MCL1 in Esophageal Squamous Cell Carcinoma (ESCC)

**DOI:** 10.3390/cancers13194766

**Published:** 2021-09-23

**Authors:** Qi Song, Herui Wang, Dongxian Jiang, Chen Xu, Jing Cui, Qi Zhang, Haixing Wang, Jie Huang, Jieakesu Su, Gen Sheng Wu, Zhengping Zhuang, Yingyong Hou

**Affiliations:** 1 Department of Pathology, Zhongshan Hospital, Fudan University, Shanghai 200032, China; qisong@fudan.edu.cn (Q.S.); jiang.dongxian@zs-hospital.sh.cn (D.J.); xu.chen@zs-hospital.sh.cn (C.X.); wang.haixing@zs-hospital.sh.cn (H.W.); huang.jie@zs-hospital.sh.cn (J.H.); su.jieakesu@zs-hospital.sh.cn (J.S.); 2 Neuro-Oncology Branch, Center for Cancer Research, National Cancer Institute, Bethesda, MD 20892, USA; herui.wang@nih.gov (H.W.); jing.cui@nih.gov (J.C.); zhangqi86@gmail.com (Q.Z.); zhengping.zhuang@nih.gov (Z.Z.); 3 Department of Oncology and Pathology, Karmanos Cancer Institute, Wayne State University School of Medicine, Detroit, MI 48201, USA

**Keywords:** esophageal squamous cell carcinoma (ESCC), nab-PTX, chemoresistance, MCL1, PP2A, LB-100

## Abstract

**Simple Summary:**

Paclitaxel-based chemotherapy has been introduced to treat esophageal squamous cell carcinoma. While its initial efficacy has been clinically established, the development of resistance is inevitable. To understand the paclitaxel resistance mechanism, we developed nanoparticle albumin-bound paclitaxel (nab-PTX)-resistant esophageal squamous cell lines from their sensitive counterparts. We found that resistant cells express higher levels of protein phosphatase 2A (PP2A), oxidative phosphorylation, and anti-apoptotic protein MCL1 than their counterparts. Therapeutically, the PP2A inhibitor LB-100 synergistically sensitized resistant esophageal squamous cells to nab-PTX both in vitro and in vivo. Therefore, our data suggest that LB-100 may potentially overcome nab-PTX resistance in a clinical setting.

**Abstract:**

Paclitaxel-based chemotherapy is a treatment option for advanced esophageal squamous cell carcinoma (ESCC). However, the development of chemoresistance leads to treatment failure, and the underlying mechanism remains elusive. We investigated the mechanisms of nanoparticle albumin-bound paclitaxel (nab-PTX) resistance by establishing three nab-PTX resistant ESCC cell lines. Proteomics analysis revealed higher oxidative phosphorylation (OXPHOS) in resistant cell line DR150 than in its parental cell line KYSE150, which is likely caused by stabilized anti-apoptotic protein MCL1. Additionally, we discovered the elevated activity of protein phosphatase 2A (PP2A), the phosphatase that dephosphorylates and stabilizes MCL1, in nab-PTX resistant cell lines. Pharmacological inhibition of PP2A with small molecule compound LB-100 decreased MCL1 protein level, caused more apoptosis in nab-PTX resistant ESCC cell lines than in the parental cells in vitro, and significantly inhibited the tumor growth of nab-PTX resistant xenografts in vivo. Moreover, LB-100 pretreatment partially restored nab-PTX sensitivity in the resistant cell lines and synergistically inhibited the tumor growth of nab-PTX resistant xenografts with nab-PTX. In summary, our study identifies a novel mechanism whereby elevated PP2A activity stabilizes MCL1 protein, increases OXPHOS, and confers nab-PTX resistance, suggesting that targeting PP2A is a potential strategy for reversing nab-PTX resistance in patients with advanced ESCC.

## 1. Introduction

Esophageal cancer (EC) is one of the most common digestive malignant tumors [1]. Due to few symptoms at the early stage and the tumor’s aggressive nature, EC is often diagnosed at an advanced stage, and recurrent disease after surgical resection is common [2]. The median survival of patients with advanced/metastatic EC treated with chemotherapy is 8–12 months [3,4,5,6]. Esophageal squamous cell carcinoma (ESCC) is the most common histological subtype of EC [1,7] and has a worse prognosis than esophageal adenocarcinoma (EAC) [8].

Paclitaxel (PTX) is a wildly used chemotherapy drug against a number of cancer types, and it functions by stabilizing the microtubule polymers and protecting them from depolymerization [9]. Nanoparticle albumin-bound paclitaxel (nab-PTX) is a solvent-free formulation of paclitaxel that is currently used as the first-line therapy for the treatment of metastatic breast cancer [10,11], local advanced/metastatic non-small cell lung cancer [12], and metastatic pancreatic cancer [13]. Emerging data has shown that nab-PTX has therapeutic benefits for various types of squamous cell carcinoma [14]. A combination of nab-PTX and cisplatin can be used as first-line therapy for metastatic ESCC [15] and as a neoadjuvant chemotherapy strategy for local advanced ESCC [16]. Although treatment with nab-PTX initially leads to improvement in the duration and quality of life for ESCC patients, a majority of these patients develop progressive disease. Thus, novel therapeutic options that overcome resistance to nab-PTX are urgently needed.

Myeloid cell leukemia-1 (MCL1) is one of the most highly amplified genes in human cancers, with amplifications observed in more than 10% of solid cancer types [17]. MCL1 is an anti-apoptotic BCL2 family member and a biomarker for anti-tubulin chemotherapeutic resistance [18]. Stabilized MCL1 is associated with paclitaxel resistance, reduced progression-free survival, and decreased overall survival in various cancers [19]. Multiple roles of MCL1 have been reported in normal cellular development and cancers. On the outer mitochondrial membrane, MCL1 antagonizes the intrinsic apoptotic pathway by regulating the permeability of the outer mitochondrial membrane, whereas a truncated isoform of MCL1 that is imported into the mitochondrial matrix facilitates mitochondrial oxidative phosphorylation (OXPHOS) [20]. Recently, the role of MCL1 in maintaining normal mitochondrial function in chemotherapeutic resistance has gained attention. A recent study showed that MYC and MCL1 could cooperatively promote paclitaxel-resistant breast cancer stem cells by increasing mitochondrial OXPHOS [21].

The MCL1 protein has a short half-life, and its rapid turnover relies on ubiquitination-mediated proteasomal degradation, a process that is dependent on the phosphorylation of MCL1 at Ser155, Ser159, and Thr163 in its PEST region [22]. Protein phosphatase 2A (PP2A) is a trimeric protein complex in which a core dimer is formed by a scaffold A subunit and a catalytic C subunit, and a whole enzyme is formed by a scaffold A subunit, a regulatory B subunit, and a catalytic C subunit. PP2A plays important roles in cell metabolism, cell cycle regulation, signal transduction, and cell proliferation [23,24]. It has been shown that PP2A selectively dephosphorylates many proteins, including the pro-survival protein MCL1 via the B subunit associated with the core enzyme [25]. Non-pharmacological inhibition of PP2A with okadaic acid, calyculin A, and PP2A/Aα knockdown all increased MCL1 Thr-163/Ser-159 phosphorylation and decreased MCL1 protein levels [26], indicating that pharmacologic inhibition of PP2A is a potential therapeutic option to disrupt stabilized MCL1 in paclitaxel-resistant tumors.

LB-100, a first-in-class small-molecule inhibitor of PP2A, has been shown to enhance the chemotherapy and radiotherapy of various cancers in preclinical studies [27]. A completed phase I trial also supported its continued development either alone or in combination with other therapies [28]. In the present study, we identified PP2A activation in nab-PTX resistant ESCC cells and investigated the possibility of restoring tumor cell sensitivity to nab-PTX by inhibiting PP2A with LB-100. Our study showed that PP2A inhibition increases ESCC sensitivity to nab-PTX by downregulating MCL1 and OXPHOS. Our data indicate that increased PP2A activity drives nab-PTX resistance in ESCC cells and suggests that targeting PP2A could be a promising strategy for overcoming nab-PTX resistance.

## 2. Results

### 2.1. OXPHOS Is Elevated in Nab-PTX Resistant ESCC Cells

**Figure 1:**
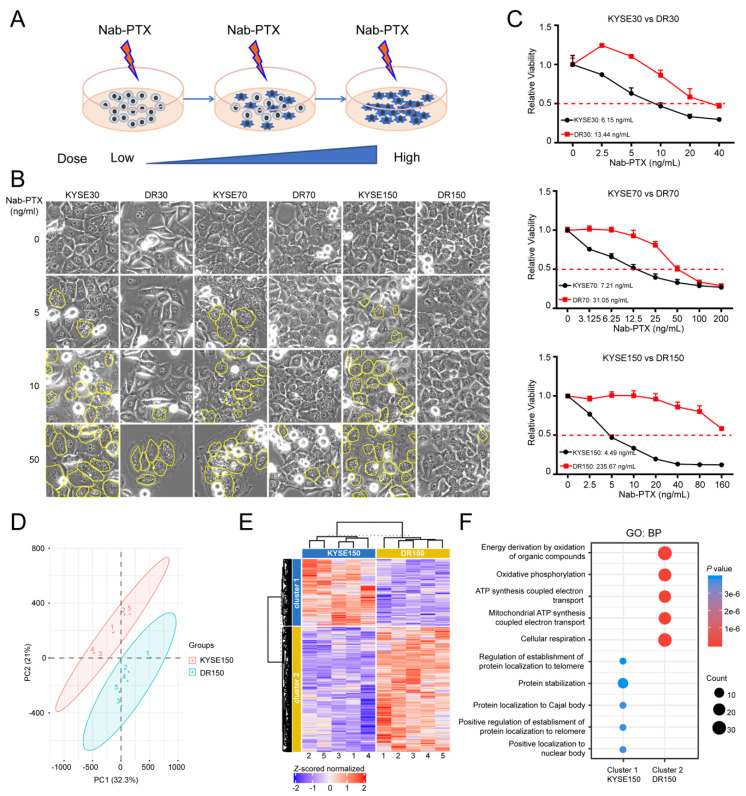
Mitochondrial oxidative phosphorylation (OXPHOS) is upregulated in nab-PTX resistant ESCC cells. (**A**) Strategy to establish nab-PTX resistant ESCC cells. Parental cells KYSE30, KYSE70 and KYSE150 were treated with gradually increasing doses of nab-PTX, starting at 1 ng/mL, for 9 months. (**B**) Cell morphology changes in KYSE30 vs. DR30, KYSE70 vs. DR70, and KYSE150 vs. DR150 cells after treatment of serial concentrations of nab-PTX for 24 h. Multi-nuclear “giant” cells were outlined in yellow. (**C**) Cell viability assay using CCK-8 confirmed higher IC50 concentrations of nab-PTX in nab-PTX resistant cell lines than in parental cells. Cells were treated with serial nab-PTX concentrations for 48 h. Cell viability at each concentration was normalized to the control group without nab-PTX treatment. IC50 concentrations were calculated by Graphpad Prism and labeled on the graphs. (**D**) PCA showed clustering of replicates and a clear separation between KYSE150 and DR150 cells. (**E**) Heatmap showed relative protein expression values of the differentially expressed proteins between KYSE150 and DR150 after unsupervised hierarchical clustering. (**F**) Geno ontology analysis of proteins significantly down-regulated (Cluster 1) and up-regulated (Cluster 2) in DR150 cells (Biological processes, BP).

In order to mimic nab-PTX resistance and explore the related mechanisms, we developed three nab-PTX resistant ESCC cell lines, named DR30, DR70, and DR150, by treating parental cells KYSE30, KYSE70, and KYSE150 cells with gradually increasing doses of nab-PTX, starting at 1 ng/mL, for 9 months (Figure 1A). The resistant cells were more resistant to nab-PTX than the corresponding parental cells. DR30, DR70 and DR150 cells continued to grow in media containing 10, 20 and 50 ng/mL nab-PTX, respectively, while the parental KYSE30, KYSE70, KYSE150 cells were very sensitive to nab-PTX treatment. Multi-nuclear “giant” cells were observed more frequently, in a dose-dependent manner, in the parental cells after nab-PTX treatment at 5, 10 and 50 ng/mL for 24 h (Figure 1B). Nab-PTX IC50 (SEM) concentrations (ng/mL) for the resistant cell lines were DR30: 13.44 (1.11), DR70: 31.05 (2.27), and DR150: 235.67 (11.98), much higher than parental cells (6.15 [0.59], 7.21 [1.17], 4.49 [2.42]), respectively (Figure 1C).

We then performed mass spectrometry with Tandem Mass Tags (TMT) technology to compare the proteomics of resistant (DR150) and parental (KYSE150) cells. In total, 3064 proteins were identified and analyzed using Proteome Discoverer software. PCA analysis showed a clustering of replicates and a clear separation between KYSE150 and DR150 cells (Figure 1D). K-means clustering of KYSE150 and DR150 cells with a total of 396 differentially expressed proteins (*p* < 0.05) were mainly divided into two clusters (Figure 1E). These differentially expressed proteins were further analyzed through pathway enrichment analysis. The top pathways of cluster 2, in which the proteins were upregulated in resistant DR150 cells, were significantly enriched with mitochondria oxidative phosphorylation (OXPHOS) (Figure 1F), suggesting elevated OXPHOS in nab-PTX resistant cells.

### 2.2. MCL1 Contributes to Nab-PTX Resistance in ESCC Cells

**Figure 2:**
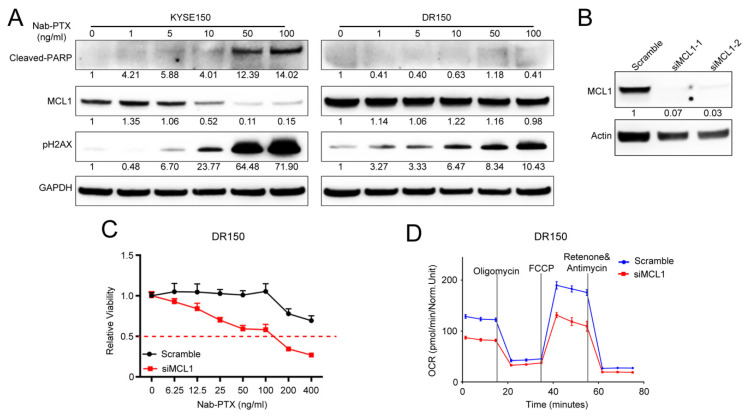
MCL1 contributes to nab-PTX resistance in ESCC. (**A**) Representative Western blot of cleaved PARP, MCL1, and γH2AX in KYSE150 and DR150 cells after 24 h treatment with titrated concentrations of nab-PTX. GAPDH was used as an internal housekeeping control. Quantified and normalized band density was labeled under each band. (**B**) Representative Western blot of MCL1 confirmed efficient MCL1 knockdown with MCL1 siRNA-1 and 2 in DR150 cells 24 h post transfection. Quantified and normalized band density was labeled under each band. (**C**) CCK-8 assay showed that MCL1 knockdown DR150 cells were more sensitive to titrated concentrations of nab-PTX than the scrambled control. (**D**) Seahorse analysis showed decreased oxygen consumption rate (OCR) in MCL1 knockdown DR150 cells compared to the scrambled control. Cells were analyzed 72 h after transfection with scramble or MCL1 siRNA.

MCL1 is a crucial regulator of apoptosis that is triggered by anti-tubulin chemotherapeutics in various malignancies, such as ovarian and breast adenocarcinomas. Aberrantly elevated expression is correlated with anti-tubulin chemotherapeutic resistance [18]. We observed more stabilized MCL1 protein in DR150 cells than in its parental KYSE150 cells in response to nab-PTX at serial concentrations (Figure 2A). Nab-PTX treatment decreased the MCL1 protein levels in KYSE150 cells in a dose-dependent manner, but not in DR150 cells (Figure 2A). Accordingly, phosphorylated H2AX (γH2AX) and cleaved PARP were significantly induced by nab-PTX in a dose-dependent manner in KYSE150 cells, while only a mild increase of γH2AX was observed in DR150 cells. The data confirm that nab-PTX induces severe DNA damage and cell apoptosis in parental KYSE150 cells but not in resistant DR150 cells, suggesting that the stabilization of MCL1 contributes to nab-PTX resistance in ESCC.

To confirm the essential role of stabilized MCL1 in nab-PTX resistance in ESCC, we knocked down MCL1 in DR150 cells (Figure 2B). Cell viability assays with CCK-8 confirmed that MCL1 knockdown DR150 cells rendered them more sensitive to nab-PTX when compared to the scrambled siRNA control (Figure 2C). Seahorse analysis also confirmed reduced oxygen consumption rate (OCR) in MCL1 knockdown DR150 cells (Figure 2D), which is consistent with the role of MCL1 in mitochondrial OXPHOS.

### 2.3. PP2A Activity Is Elevated in Nab-PTX Resistant ESCC Cells

Since protein phosphatase 2A (PP2A) was reported to dephosphorylate MCL1 at Ser159 and Thr163 in its PEST region and prevent its rapid degradation in lymphoma cells [26], we hypothesized that PP2A may participate in MCL1 stabilization during nab-PTX resistance in ESCC. To determine if PP2A is involved in ESCC progression, we analyzed Kaplan-Meier Plotter datasets (http://kmplot.com, accessed date: 8 November 2019) and found that the expression level of PPP2CA, the major catalytic subunit of PP2A, was negatively correlated with the overall survival rate of esophageal cancer (Supplementary Figure S1A,B). High PPP2CA expression in tumors leads to shorter overall survival in both esophageal squamous cell carcinoma (ESCC) and esophageal adenocarcinoma (EAC) patients (*p* = 0.065 and 0.0085, respectively, log-rank test), suggesting the important role of PP2A in ESCC progression.

**Figure 3:**
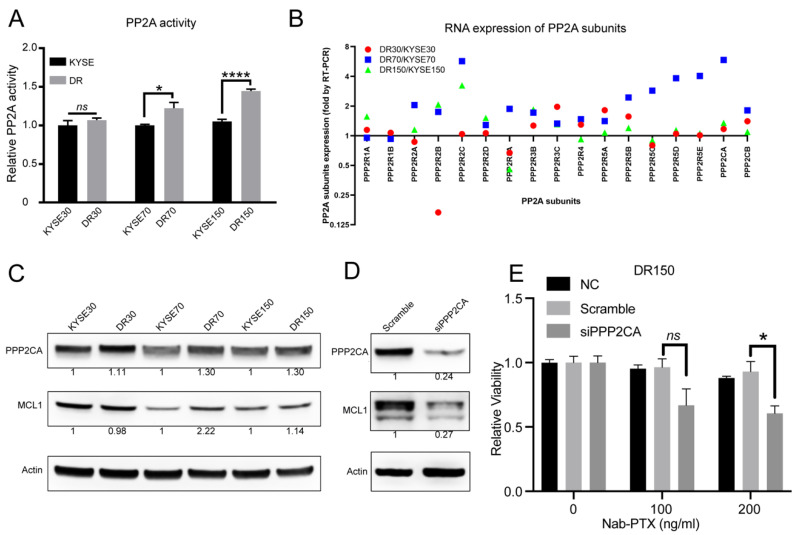
Elevated PP2A activity in nab-PTX resistant ESCC cells. (**A**) PP2A activity of parental and nab-PTX resistant cells was measured using a PP2A immunoprecipitation (IP) phosphatase assay kit. PP2A activity in resistant cell lines was normalized to the corresponding parental cell line. (**B**) Real-time RT PCR for all PP2A subunit genes. Gene expression level in resistant cell lines was normalized to that in the corresponding parental cell line. (**C**) Representative Western blot analysis of PPP2CA and MCL1 in all cell lines. β-ACTIN serves as an internal control. Quantified and normalized band density was labeled under each band. (**D**) Western blot confirmed decreased MCL1 protein in PPP2CA knockdown DR150 cells. (**E**) Cell viability assay showed that PPP2CA knockdown DR150 cells were more sensitive to nab-PTX when treated at 200 ng/mL. DR150 cells were transfected with scramble or PPP2CA siRNA for 24 h and then treated with vehicle control or nab-PTX (100 or 200 ng/mL) for 48 h. DR150 cells without any transfections (DR150-NC) were also included as a control group.

To confirm whether PP2A is involved in nab-PTX resistance in ESCC, we compared the PP2A activity in the three established nab-PTX resistant cell lines with their parental cells. Compared to their corresponding parental cells, PP2A activity was significantly elevated in DR70 and DR150 cells (Figure 3A). We also observed a trend of increased PP2A activity in DR30 cells, which was the cell line that was least resistant to nab-PTX among all three resistant cell lines (Figure 3A). We then measured the mRNA expression levels of all PP2A subunits using real-time RT-PCR in all three pairs of cell lines. We found that the mRNA levels of PP2A regulatory subunits (PPP2R2C, PPP2R2D, PPP2R3B, PPP2R3C, PPP2R5A, PPP2R5B, PPP2R5D) and catalytic subunits (PPP2CA, PPP2CB) were consistently higher in the nab-PTX resistant cells as compared to the corresponding parental cells (Figure 3B). Western blot also confirmed increased PPP2CA protein levels in all three nab-PTX resistant cell lines in comparison to the corresponding parental cells (Figure 3C). MCL1 protein levels were also increased in DR70 and DR150 cells (Figure 3C). To further confirm the essential roles of PP2A in MCL1 upregulation and nab-PTX resistance, we knocked down PPP2CA in DR150 cells using siRNA (Figure 3D). Compared to the scrambled control, PPP2CA-knockdown DR150 cells showed decreased MCL1 protein level and reduced cell viability after treatment with nab-PTX at 200 ng/mL for 48 h (Figure 3D,E). In summary, these data confirm the critical roles of PP2A in stabilizing MCL1 and conferring nab-PTX resistance.

### 2.4. Pharmacological Inhibition of PP2A Inhibits the Growth of Nab-PTX Resistant ESCC Xenografts

**Figure 4:**
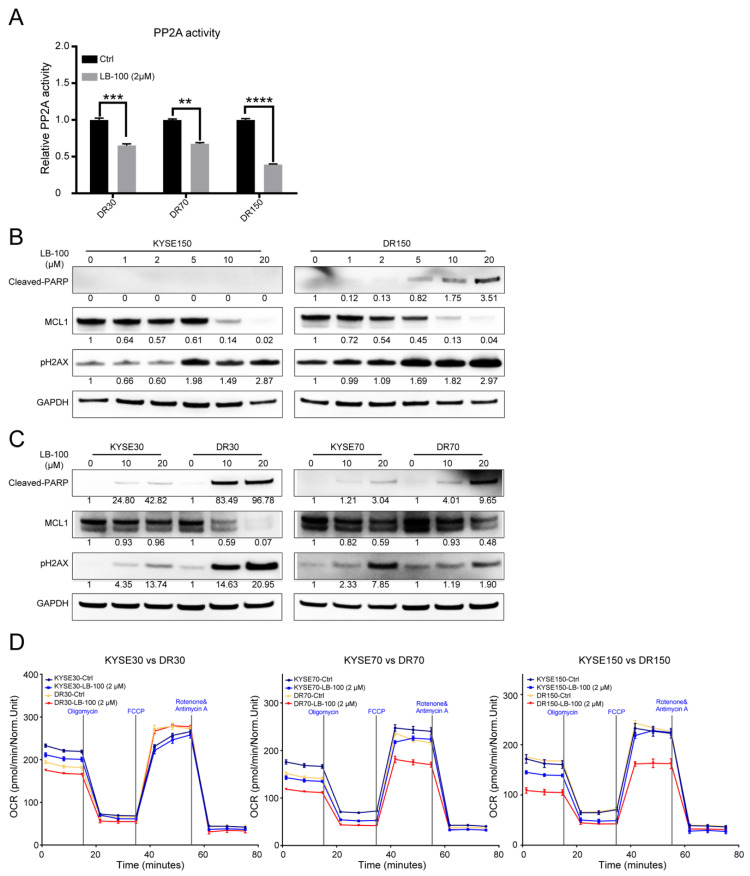
LB-100 decreased PP2A activity and promoted MCL1 degradation in nab-PTX resistant cells. (**A**) PP2A activity was decreased in nab-PTX resistant cell lines treated with LB-100 at 2 μM for 6 h. Data are shown as mean ± SEM. ** *p* < 0.01, *** *p* < 0.001, **** *p* < 0.0001, two-tailed Student’s *t*-test. (**B**,**C**) Representative Western blot analysis of cleaved-PARP, MCL1, and γH2AX in KYSE150 vs. DR150 cells (B), KYSE30 vs. DR30 cells, and KYSE70 vs. DR70 cells (**C**) 24 h post LB-100 treatment at serial concentrations. GAPDH served as an internal control. Quantified and normalized band density was labeled under each band. (**D**) Seahorse analysis in KYSE30 vs. DR30, KYSE70 vs. DR70, and KYSE150 vs. DR150 cells treated with vehicle control or 2 μM LB-100 for 6 h.

LB-100 is a first-in-class small-molecule inhibitor of PP2A that has already passed the Phase-I clinical trial with favorable outcomes [28]. Based on the elevated PP2A activity in resistant cell lines, we first evaluated the potential therapeutic efficacy of pharmacologically inhibiting PP2A with LB-100 in vitro. PP2A activity was decreased by 40–60% in nab-PTX resistant cell lines treated with LB-100 at 2 μM for 6 h (Figure 4A). LB-100 treatment also decreased MCL1 protein levels in a dose-dependent manner, and MCL1 protein levels seemed more sensitive to LB-100 treatment in nab-PTX resistant cell lines than in the parental cells (Figure 4B,C). Accordingly, DNA damage marked by γH2AX and cell apoptosis marked by cleaved PARP were also more efficiently induced by LB-100 treatment in the resistant cell lines (Figure 4B,C). Seahorse analysis also confirmed decreased OCR in LB-100-treated DR70 and DR150 cells, but not in the parental KYSE70 or KYSE150 cells (Figure 4D).

**Figure 5:**
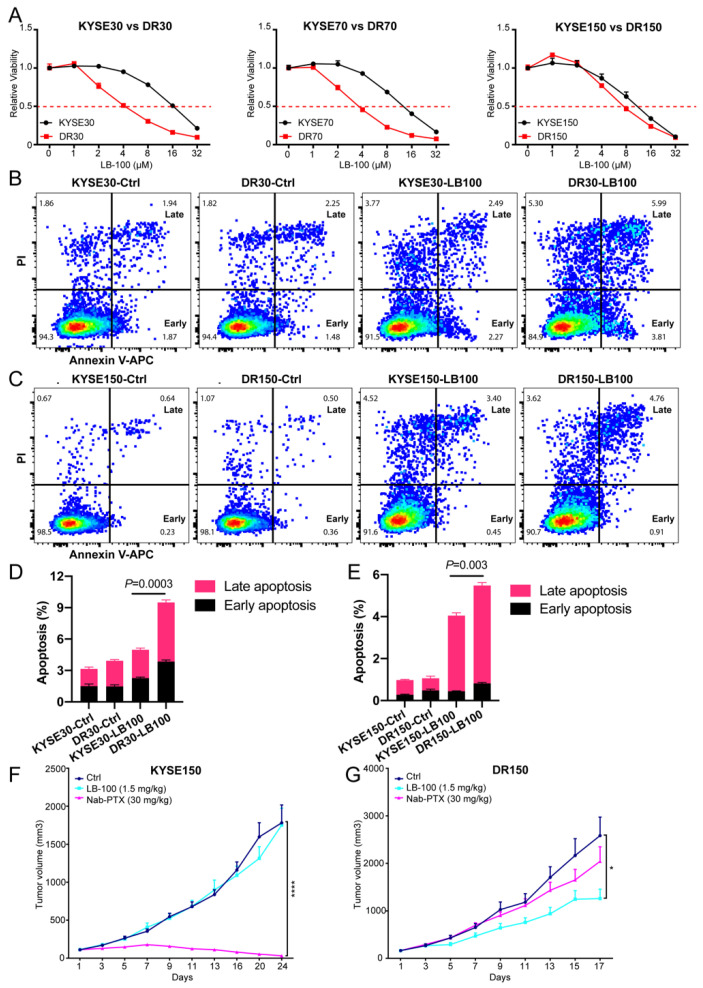
Nab-PTX resistant cells are more sensitive to LB-100 treatment than parental cells both in vitro and in vivo. (**A**) CCK-8 assay of nab-PTX resistant cells vs. parental cells after treatment with titrated concentrations of LB-100. For each cell line, cell viability at each LB-100 concentration was normalized to the control group without LB-100 treatment. Data are shown as mean ± SEM. (**B**,**D**) LB-100 treatment at 2 μM for 48 h induced more cell apoptosis in DR30 cells than in parental KYSE30 cells. (**B**): Representative dot plot images of control and LB-100-treated KYSE30 and DR30 cells showing population gating for early apoptosis (Early) and late apoptosis (Late). (**D**): Quantitative analysis results of early and late apoptotic cells in KYSE30 and DR30 cells with or without LB-100 treatment. *p* value was analyzed with a two-tailed Student’s *t*-test between the total apoptotic cells of LB100-treated KYSE30 cells and DR30 cells. (**C**,**E**) LB-100 treatment at 2 μM for 48 h induced more cell apoptosis in DR150 cells than in parental KYSE150 cells. (**F**,**G**) Tumor growth curves for KYSE150 xenograft mouse model (**F**) and DR150 xenograft mouse model (**G**). Tumor-bearing mice from each model were treated in three groups: intraperitoneal (i.p.) injection with vehicle control every other day (*n* = 10), i.p. injection with LB-100 every other day (1.5 mg/kg, *n* = 10), and tail vein injection with nab-PTX every four days (30 mg/kg, *n* = 10). Data are shown as mean ± SEM. *p* values were calculated using a two-tailed Student’s *t*-test at indicated time points. * *p* < 0.05; **** *p* < 0.0001.

Next, we checked if LB-100 is more cytotoxic to nab-PTX resistant cells than to parental cells. Notably, LB-100 IC50 (SEM) concentrations in DR30, DR70 and DR150 cells (4.58 [0.54], 4.12 [0.87], 5.23 [0.90], respectively) were significantly lower than their parental cells (16.03 [0.09], 12.66 [0.46], 9.81 [0.40], respectively) (Figure 5A). We also quantified cell apoptosis using Annexin V/PI double staining for flow cytometry in KYSE30 vs. DR30 and KYSE150 vs. DR150 cells after LB-100 treatment at 2 uM for 48 h. Consistently, LB-100 treatment induced more early apoptosis (Annexin V+ PI-) and late apoptosis (Annexin V+ PI+) in both DR30 and DR150 cells, in comparison to KYSE30 and KYSE150 cells, respectively (Figure 5B–E).

We then validated LB-100 efficacy in vivo on mouse xenograft models using KYSE150 and DR150 cells. When the mean tumor volume reached about 100 mm^3^, we randomized the tumor-bearing mice into three groups: vehicle control, LB-100 treatment (1.5 mg/kg every other day), and nab-PTX treatment (30 mg/kg every four days). In KYSE150 xenograft models, nab-PTX efficiently regressed tumor growth, but LB-100 alone (1.5 mg/kg) exhibited no effects (Figure 5F). Conversely, in nab-PTX resistant DR150 xenograft models, nab-PTX treatment failed to inhibit DR150 tumor growth, but LB-100 significantly inhibited the tumor growth (Figure 5G). These data confirm that compared to parental cells, nab-PTX-resistant cells are more sensitive to LB-100 both in vitro and in vivo.

### 2.5. Pharmacological Inhibition of PP2A Resensitizes Nab-PTX Resistant ESCC to Nab-PTX

**Figure 6:**
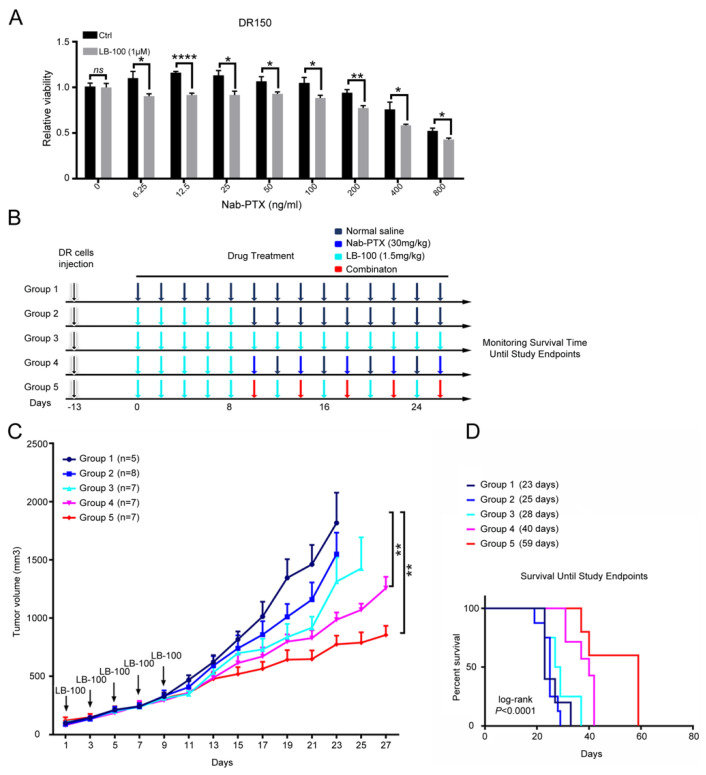
LB-100 treatment re-sensitizes DR150 cells and xenograft models to nab-PTX. (**A**) CCK-8 assay showed decreased cell viability in LB-100 pretreated DR150 cells under titrated nab-PTX treatment. DR150 cells were pretreated with LB-100 (1 μM) for three days before nab-PTX treatment. CCK-8 assay was performed two days after nab-PTX treatment. The cell viability of each group was normalized to the control DR150 cells without LB-100 pretreatment. * *p* < 0.05, ** *p* < 0.01, **** *p* < 0.0001. (**B**) Experimental design for the DR150 xenograft mouse model. Normal saline and LB-100 (1.5 mg/kg) were administered intraperitoneally every other day and nab-PTX (30 mg/kg) was administrated by tail vein injection every four days. (**C**) Tumor growth curves of each group. Group size (n) was labeled in the figure. Data are shown as mean ± SEM. *p* values were calculated using a two-tailed Student’s *t*-test. ** *p* < 0.01. (**D**) Overall survival curves of all five groups (*p* < 0.0001, log-rank test).

Lastly, we explored the possibility of PP2A inhibition with LB-100 in resensitizing nab-PTX resistant ESCC cells to nab-PTX. We pretreated DR150 cells with LB-100 at a low dose (1 μM) for three days and then checked the cell viability after nab-PTX treatment. Compared to DR150 cells without LB-100 pretreatment, pretreated DR150 cells exhibited lower cell viability after treatment with titrated nab-PTX (Figure 6A), indicating that LB-100 partially resensitized nab-PTX resistant ESCC cells to nab-PTX.

We then tested this hypothesis in vivo using nab-PTX resistant xenograft models. In DR150 xenograft mouse models, tumor-bearing mice were first treated with normal saline (Group 1) or LB-100 (1.5 mg/kg) every other day for 5 doses. LB-100 treated mice were then assigned into 4 groups: normal saline (Group 2), LB-100 (1.5 mg/kg every other day, Group 3), nab-PTX (30 mg/kg every four days, Group 4), and LB-100 and nab-PTX combination (Group 5) (Figure 6B). We found that LB-100 pre-treatment mildly inhibited tumor growth (Figure 6C, Group 2 vs. Group 1). Nab-PTX treatment significantly inhibited tumor growth (Figure 6C, Group 4). Since nab-PTX failed to inhibit DR150 tumor growth by itself without LB-100 pretreatment (Figure 5G), this data indicates that LB-100 pretreatment partially resensitized the resistant xenografts to nab-PTX. The LB-100 and nab-PTX combination group showed the slowest tumor growth (Figure 6C, Group 5), suggesting a synergistic effect between LB-100 and nab-PTX. We also monitored these tumor-bearing mice until the study endpoints (Figure 6D). Consistent with the tumor growth curve, Group 4 showed significantly longer survival than Group 1 (*p* = 0.003; overall survival: 40 vs. 23 days) and Group 2 (*p* = 0.0001; overall survival: 40 vs. 25 days). Group 5 showed the longest survival time with overall survival of 59 days (*p* = 0.0015 and 0.0007, compared to Group 1 and 2, respectively; Figure 6D), which is consistent with the observation that this group had the slowest tumor growth (Figure 6C). We obtained similar results in DR70 xenograft mouse models (Supplementary Figure S2). LB-100 pretreatment increased the sensitivity of DR70 xenografts to follow-up nab-PTX treatment. Combined treatment of LB-100 and nab-PTX led to the slowest tumor growth in DR70 xenografts, confirming the synergistic effect between LB-100 and nab-PTX in vivo.

## 3. Discussion

Nab-PTX is an effective treatment against advanced and metastatic ESCC, but the emergence of drug resistance limits its therapeutic benefit, and the underlying mechanism is not fully understood. Herein, we identified elevated PP2A activity in established nab-PTX resistant ESCC cell lines and proved that PP2A likely promotes nab-PTX resistance by stabilizing MCL1 protein. We further proved that pharmacological inhibition of PP2A by LB-100 is more cytotoxic to nab-PTX resistant cells than to parental cells, and LB-100 treatment partially re-sensitizes resistant cells to nab-PTX both in vitro and in vivo. Therefore, selective PP2A inhibitor LB-100 is a promising therapeutic option for the treatment of nab-PTX resistant ESCC.

MCL1 has been suggested to play an essential role in resistance to anti-microtubule chemotherapeutics [18]. However, it is not clear whether MCL1 contributes to nab-PTX resistance in ESCC. In DR150 cells, MCL1 protein levels are increased (Figure 3C) and are stabilized upon nab-PTX treatment (Figure 2A). MCL1 knockdown also decreased oxygen consumption rate and partially restored nab-PTX sensitivity in DR 150 cells (Figure 2C,D). Our findings confirm the contribution of MCL1 to nab-PTX resistance in ESCC, consistent with other cancer types [21].

MCL1 protein levels are tightly regulated, and its rapid degradation relies on phosphorylation-dependent ubiquitination by FBW7 and subsequent proteasomal degradation [18]. ERK-mediated phosphorylation of MCL1 at Thr163 in the PEST region was shown to stabilize MCL1 protein [17]. Consistently, non-pharmacological inhibition of PP2A prevents MCL1 protein dephosphorylation at Thr-163/Ser-159 in the PEST region and dramatically reduces MCL-1 protein levels in MCL1-amplified lymphoma cells [26]. Our study herein demonstrated that stabilized MCL1 protein is dependent on elevated PP2A activity in nab-PTX resistant ESCC cell lines. We exhibited increased gene expression of most PP2A subunits and elevated PP2A activity in nab-PTX resistant ESCC cell lines (Figure 3A–C). Knockdown of PPP2CA, the major catalytic subunit of PP2A, significantly decreased MCL1 protein levels and partially restored cell sensitivity to nab-PTX in DR150 cells (Figure 3D,E). These results suggest that PP2A participates in the development of nab-PTX resistance in ESCC cells by stabilizing MCL1.

Our findings also suggest that PP2A serves as a potential therapeutic target for nab-PTX resistance in ESCC. We confirmed this hypothesis by utilizing a first-in-class small molecule inhibitor of PP2A, LB-100, that has completed Phase-I clinical trials with favorable outcomes [28]. Nab-PTX resistant cells are more sensitive to LB-100 than their parental cells. Specifically, LB-100 treatment (1) promoted faster degradation of MCL1 and accordingly decreased OCR in resistant cells (Figure 4B–D); (2) induced more apoptosis in nab-PTX resistant cells (Figure 4B,C, Figure 5A–E); and (3) efficiently inhibited the tumor growth in nab-PTX resistant xenografts (DR150) but not in nab-PTX sensitive xenografts (KYSE150) (Figure 5F,G). These results indicate that nab-PTX resistant cells are more dependent on PP2A for survival. Mass spectrometry with Tandem Mass Tags compared the proteomics of DR150 and KYSE150 cells at the molecular level and revealed significantly increased levels of proteins that participate in the OXPHOS pathway in DR150 cells (Figure 1D–F). In Figure 4D, we did see a trend of OCR increase in DR150-Ctrl cells compared to KYSE150-Ctrl cells, but the Seahorse assay may not be sensitive enough to reveal small OCR changes between these two cell lines under our current experimental conditions.

Consistent with the correlated roles of PP2A and MCL1 in nab-PTX resistance, we further proved that LB-100 pretreatment re-sensitized nab-PTX resistant cells to nab-PTX both in vitro and in vivo (Figure 6, supplementary Figure S2). Synergistic effects of LB-100 and nab-PTX treatment were also observed in mouse nab-PTX resistant xenograft models. Therefore, we hypothesize that LB-100 can be used as a chemosensitizer to enhance the efficacy and eliminate/delay the resistance of nab-PTX. Based on the shared mechanisms in paclitaxel resistance among different cancers, our findings may also be applicable in other cancer types. The significance of our preclinical observations warrants further validation in clinical trials.

In summary, this is, to the best of our knowledge, the first report that PP2A activity is increased in nab-PTX resistant ESCC cell lines. The underlying mechanisms include increased and stabilized MCL1 and enhanced OXPHOS in the resistant cells. Pharmacological inhibition of PP2A by LB-100 partially restored nab-PTX sensitivity in ESCC via decreasing MCL1 protein level and OXPHOS. These findings provide pre-clinical insights for future clinical trials that combine nab-PTX and LB-100 treatment in ESCC patients.

## 4. Materials and Methods

### 4.1. Cell Lines

KYSE30, KYSE70, and KYSE150 were kindly provided by Dr. Pan from the University of Texas at Arlington. Cells were cultured in a mixture of RPMI 1640 and F12 medium (1:1), supplemented with 10% fetal bovine serum, and 1% penicillin/streptomycin. All cells were confirmed to be free of mycoplasma contamination using a MycoAlert Mycoplasma Detection Kit (Lonza, Walkersville, MD, USA).

### 4.2. Reagents

LB-100 was provided by Lixte Biotechnology, East Setauket, NY, under NCI M-CRADA # 03094. Nab-PTX was purchased from the Celgene Corporation (Summit, NJ, USA).

### 4.3. Development of nab-PTX Resistant Cell Lines and Cell Viability Assay

Resistant cells were developed by culturing KYSE30, KYSE70 and KYSE150 ESCC cell lines with increasing doses of nab-PTX continuously over 9 months. Cell viability assay was performed with Cell Counting Kit-8 (CCK-8) (Dojindo Molecular Technologies, Inc, Rockville, MD, USA), according to the manufacturer’s instructions. Six replicates were used for each group. Relative cell viability was obtained by normalizing to the indicated control group.

### 4.4. Proteomics Sample Preparation and LC-MS-Based Proteomics

Five biological replicates of KYSE150 and DR150 cell lines were used for quantification and comparison. Samples were digested with trypsin. The lysates of each sample were labeled with one of the 0.2 mg TMT 10Plex reagents (ThermoFisher Scientific, Pleasanton, CA, USA). Labeled samples were mixed and then fractionated with 2D separation. Each fraction was used to perform an LC-MS/MS run. The raw data were used to generate the global protein quantitation results. Proteome Discoverer software was used for the database analysis and the ratio calculation. One replicate sample (#1) of KYSE150 was used as a reference. 

### 4.5. Western Blot Analysis

Cells were lysed with RIPA buffer (Thermo Scientific) containing 10 mM sodium fluoride, 1 mM sodium orthovanadate, 25 mM sodium glycerophosphate, 1× protease inhibitor cocktail (Roche diagnostics, Indianapolis, IN, USA) and 1 mM PMSF. Lysates (20 μg) of each sample ran in SDS-PAGE gels and were then transferred to nitrocellulose membranes. The membranes were blocked and then incubated with primary antibodies at 4 °C overnight, followed by incubation with HRP-conjugated anti-rabbit or anti-mouse secondary antibodies for 1 h at room temperature. Western blot bands were detected by enhanced chemiluminescence substrate (R1004, Kindle Biosciences, Greenwich, CT, USA) and imaged by KwikQuant Imager (D1001, Kindle Biosciences). Primary antibodies used in this study: cleaved PARP (CST #5625, Cell Signaling Technology Inc, Danvers, MA, USA), MCL1 (CST #39224), γH2AX (CST #9718), PPP2CA (Millipore #05-421, St. Louis, MO, USA), GAPDH (Millipore #MAB374), β-ACTIN (CST #3700).

### 4.6. PPP2CA, and MCL1 Knockdown with siRNA

PPP2CA, MCL1, and control siRNAs were purchased from IDT. DR150 cells were seeded in 6-well plates 24 h prior to transfection. Lipofectamine RNAiMAX (ThermoFisher Scientific) was dissolved in Opti-MEM for 5 min at room temperature and then mixed with Opti-MEM containing siRNA for 15 min at room temperature. The final concentration of siRNA was 20 nM. The knockdown efficiency was confirmed by western blot.

### 4.7. Seahorse Analysis (XF Cell Mito Stress Test)

The mitochondrial respiratory capacity was determined using an XF Cell Mito Stress Test Kit (Agilent Technologies, Santa Clara, CA, USA). Cells (20,000 cells per well) were seeded in the XF Cell Culture Microplate and incubated for 24 h at 37 °C. Pretreated cells were then incubated with the base medium containing 2mM L-glutamine, 10 mM sodium pyruvate, and 10 mM glucose for 1 h prior to assay. The oxygen consumption rate (OCR) was measured by an XFe96 extracellular flux analyzer (Agilent Technologies) with a sequential injection of 1mM oligomycin A, 0.5 mM FCCP, and 0.5 mM rotenone/antimycin A.

### 4.8. PP2A Activity Assay

Cells were grown to about 80% confluence in 100 mm dishes and treated as indicated. LB-100 was given 6 h prior to PP2A activity testing. Cells were washed twice with cold TBS (pH 7.4) and lysed in 0.3% NP-40 (ThermoFisher Scientific) supplemented with protease inhibitor (Roche) for 30 min on ice. Cell lysates were sonicated and then centrifuged at maximum speed for 15 min. Supernatants containing 50 μg of total cellular protein were assayed with the PP2A Immunoprecipitation Phosphatase Assay Kit (Millipore, 17-313) according to the manufacturer’s instructions. Experiments were performed in triplicate, and PP2A activity was normalized with the indicated control group. Data are presented as mean ± SEM.

### 4.9. Cell Apoptosis Assay

Cells were dissociated with Trypsin and incubated with 1× Annexin V binding buffer containing Annexin V-APC (BD) for 30 min at room temperature in the dark. Cells were then incubated with propidium iodide (PI, Sigma, St. Louis, MO, USA) for 10 min at room temperature in the dark before flow cytometry analysis.

### 4.10. Animal Studies

Animal studies were approved by the Animal Care and Use Committee of NCI. A total of 2 million cells per mouse were injected subcutaneously in the flank of immunodeficient NSG mice. Mice were randomized into indicated groups when mean tumor volumes reached 100 mm^3^ and n ≥ 5 mice per group were used. Tumor volumes were assessed every other day by standard caliper measurement (V = L × W^2^/2). The study endpoint was defined according to the following criteria: (1) tumor volume exceeds 2000 mm^3^, (2) tumor diameter exceeds 20 mm, (3) severe non-healing skin necrosis over the tumor, (4) moribund behavior. Mice will be euthanized when they meet any one of the listed conditions.

### 4.11. Statistical Analysis

All analyses were performed with GraphPad Prism 8 or R (3.6.2). For proteomic analyses, the protein groups output table was used. Data were normalized. To extract differentially expressed proteins (DEPs) between KYSE150 and DR150, a two-sample Student’s *t*-test was performed with a *p* value threshold of 0.05. A ComplexHeatmap package was used for hierarchical clustering. GO enrichment analysis by the clusterProfiler package was used for DR150 vs KYSE150 cells. Data were presented as mean ± SEM. All experiments were conducted at least twice with representative data shown. Statistical tests were also indicated in the texts where *p* values were provided.

## 5. Conclusions

To the best of our knowledge, this is the first report that PP2A activity is increased in nab-PTX resistant ESCC cells. PP2A likely promotes nab-PTX resistance by stabilizing MCL1. Pharmacological inhibition of PP2A by LB-100 partially restored nab-PTX sensitivity and inhibited nab-PTX resistant xenograft growth alone or in combination with nab-PTX, supporting PP2A as a novel therapeutic target against nab-PTX resistant ESCC.

## Data Availability

The data presented in this study are available on request from the corresponding authors. The data are not publicly available due to privacy restrictions.

## Supplementary Materials

The following are available online at https://www.mdpi.com/article/10.3390/cancers13194766/s1. Figure S1: PPP2CA expression is negatively correlated with overall survival of esophageal cancer. Figure S2: LB-100 treatment re-sensitized DR70 xenograft models to nab-PTX. Original western blot images are shown in the supplementary materials.
