# Supplementary material for: Pharmacological Inhibition of PP2A Overcomes Nab-Paclitaxel Resistance by Downregulating MCL1 in Esophageal Squamous Cell Carcinoma (ESCC)
Source: Cancers (Basel). 2021 Sep 23;13(19):4766. doi: 10.3390/cancers13194766 (PMC8507542; doi:10.3390/cancers13194766)

Original western blot images

Full unedited blots for Figure 2A

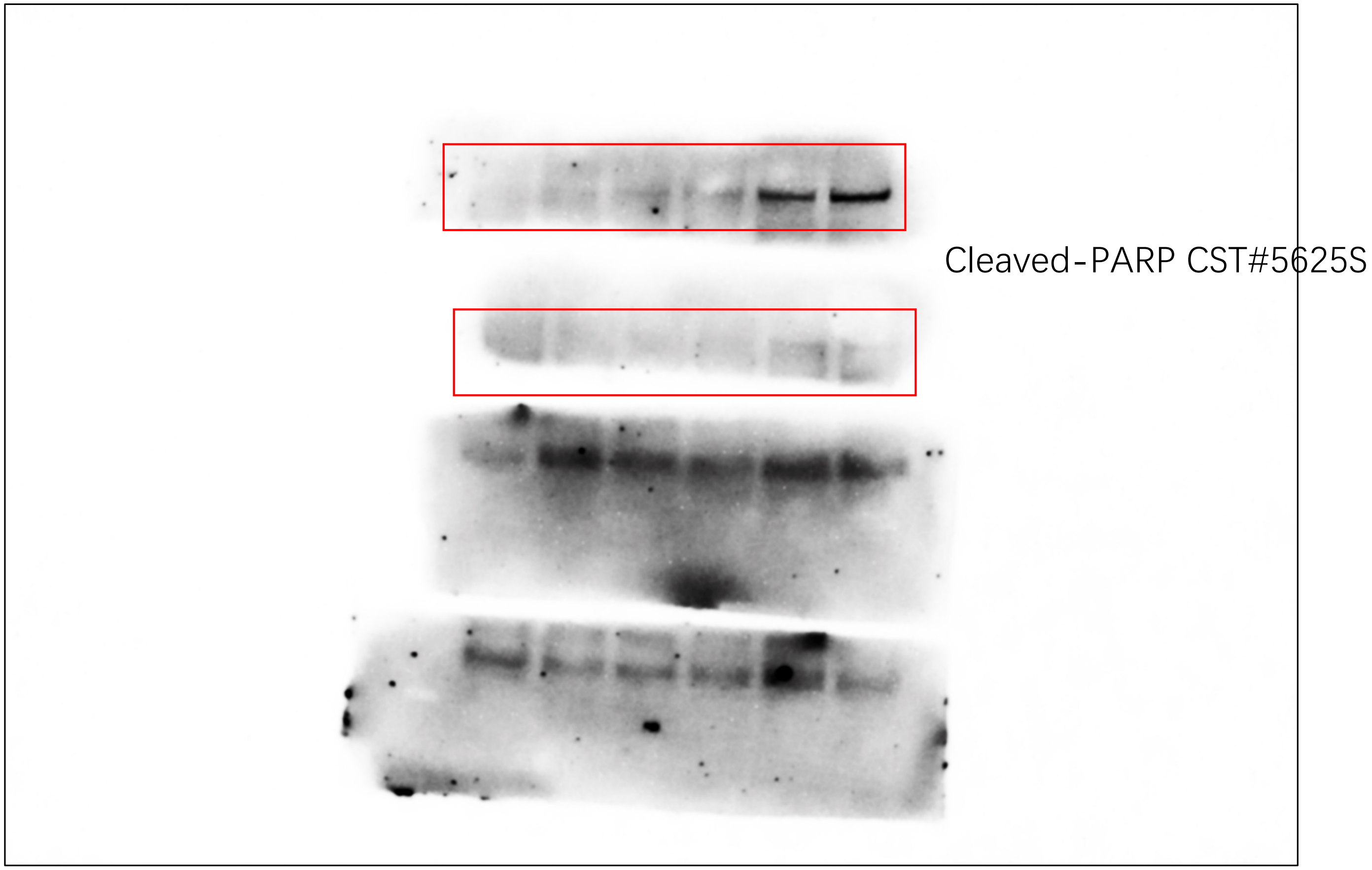

Full unedited blots for Figure 2A

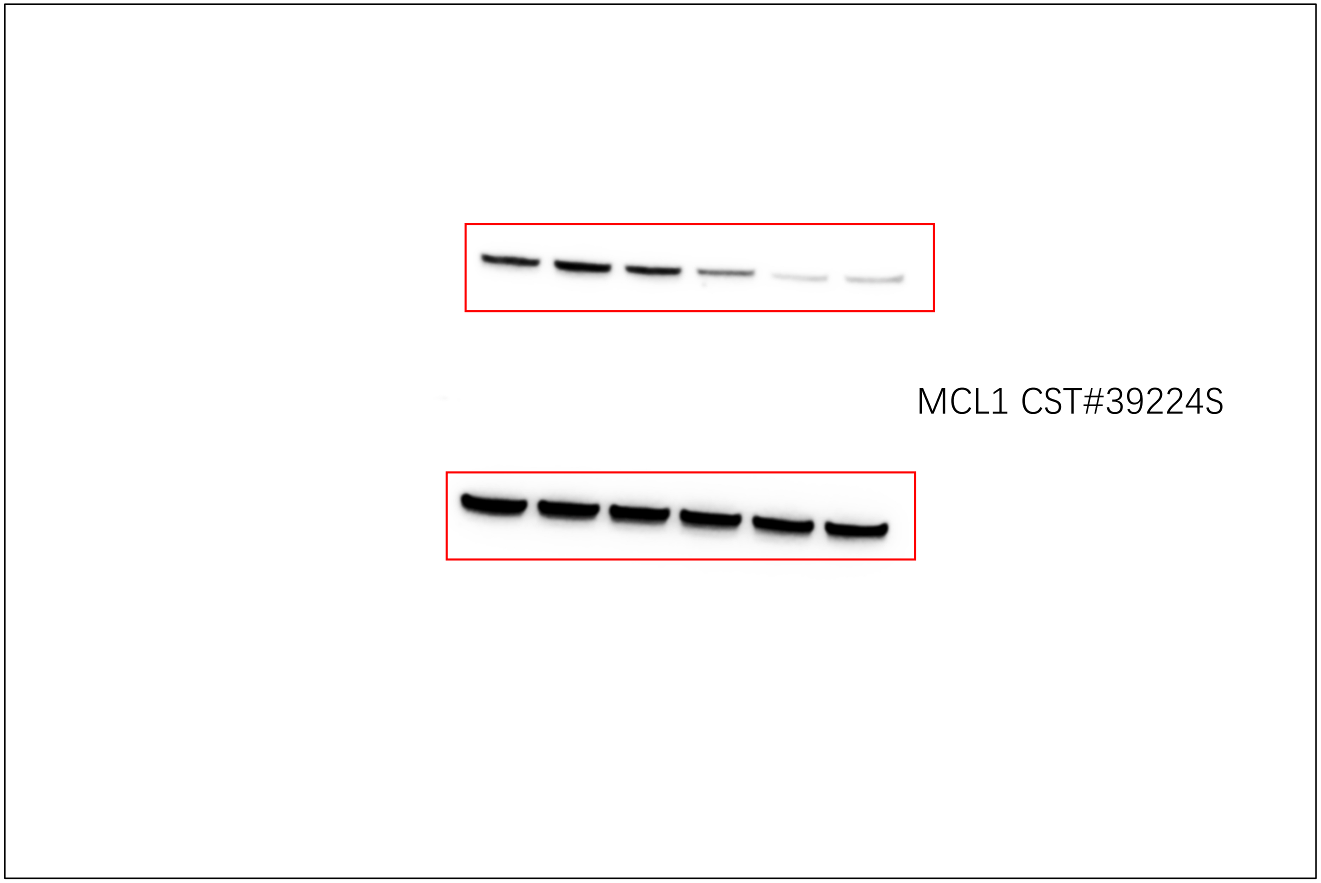

Full unedited blots for Figure 2A

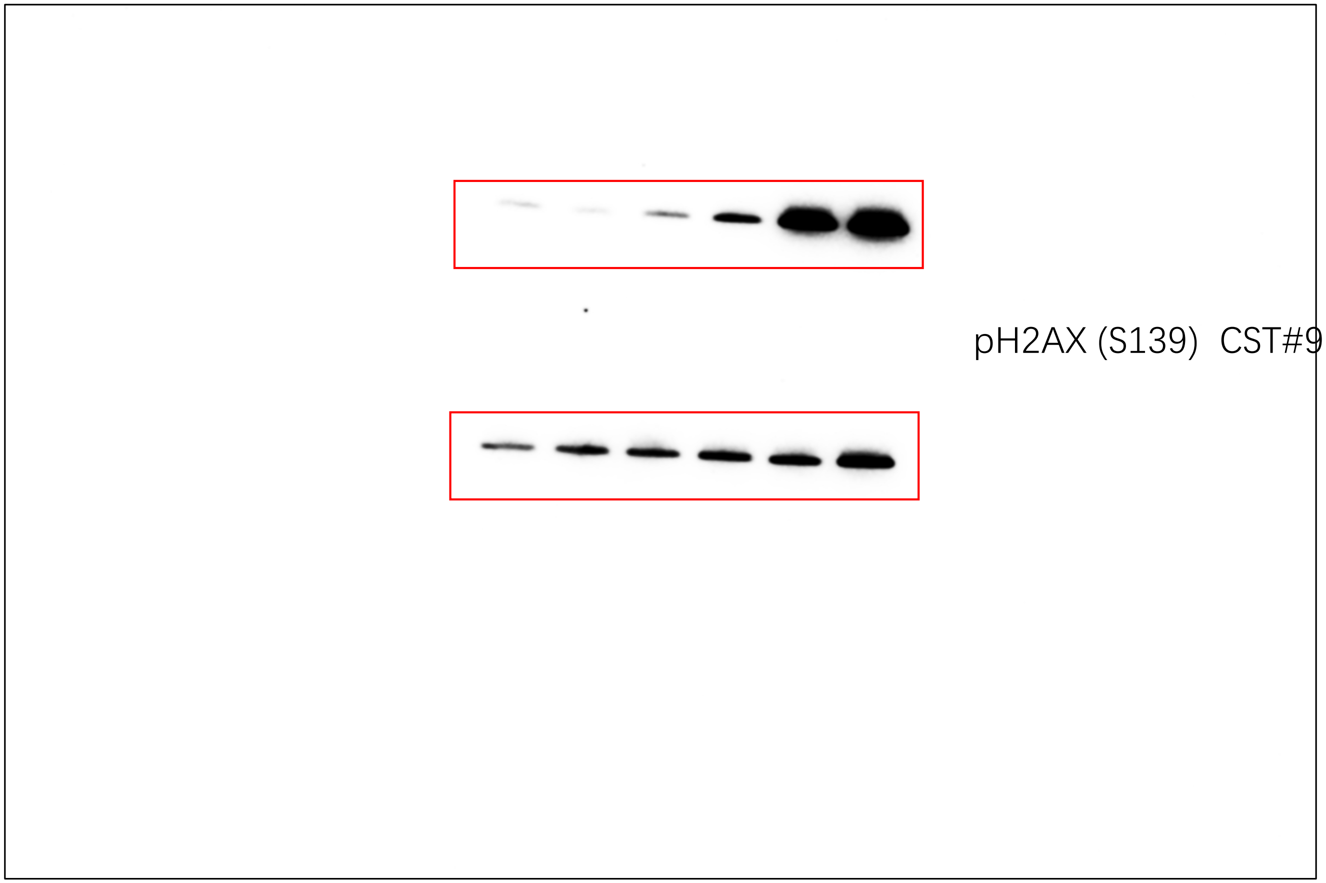

pH2AX (S139) CST#9718S

Full unedited blots for Figure 2A

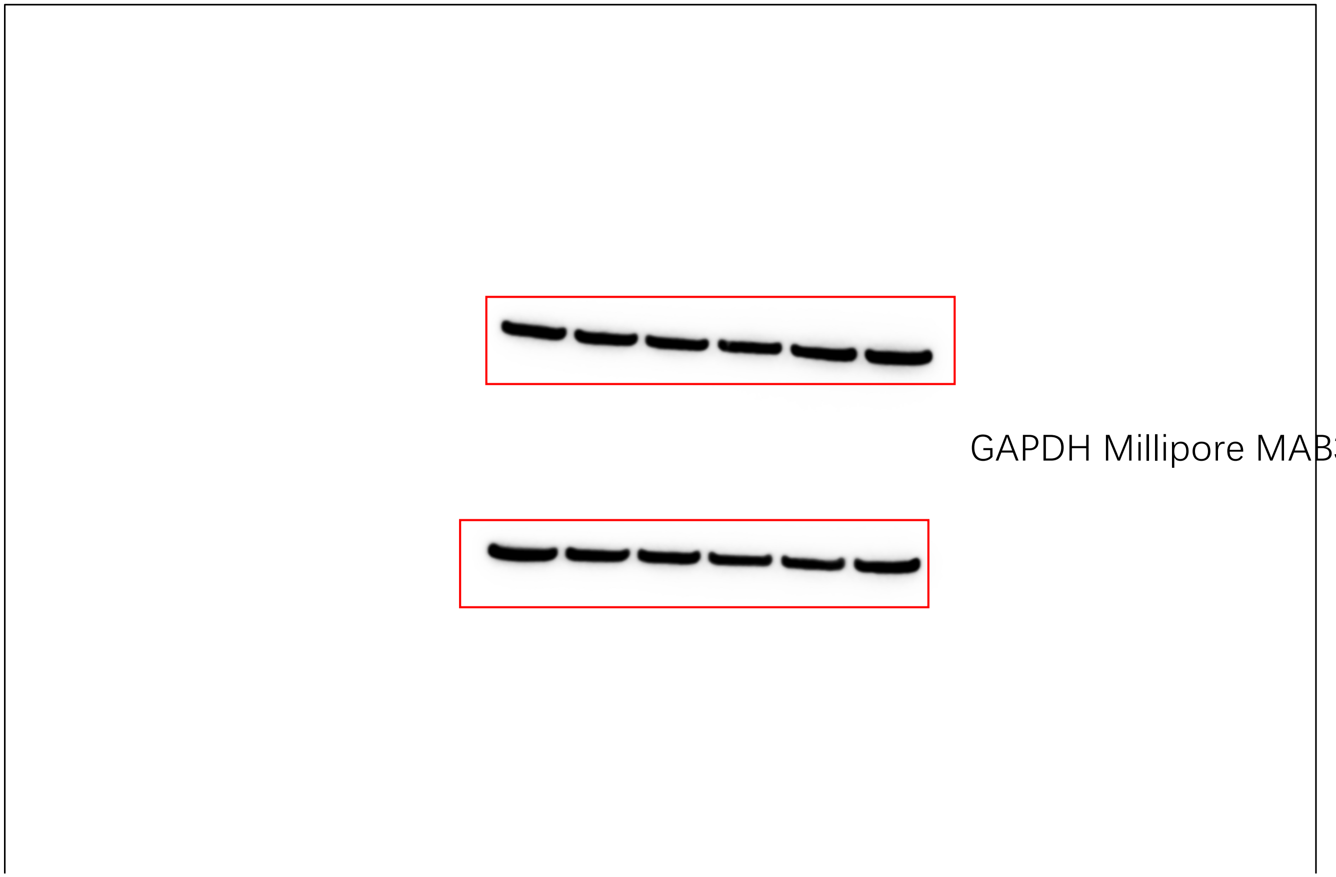

GAPDH Millipore MAB374

Full unedited blots for Figure 2B

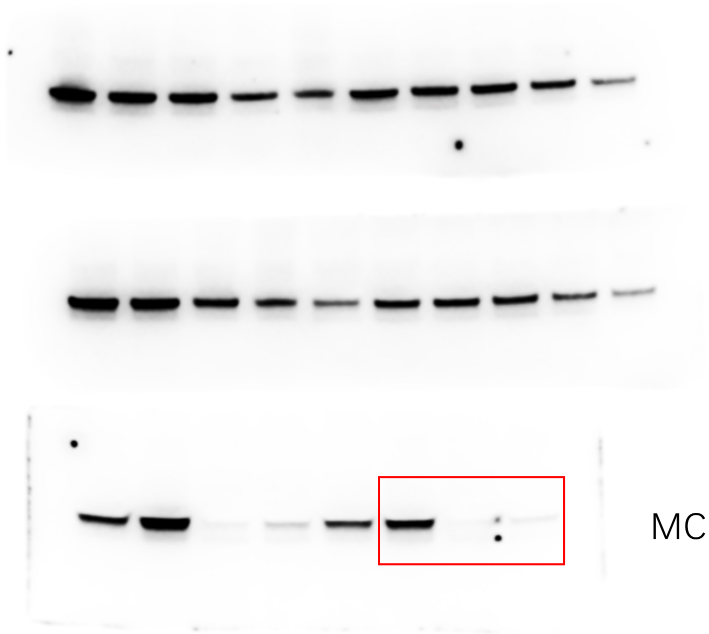

MCL1 CST#39224S

Full unedited blots for Figure 2B

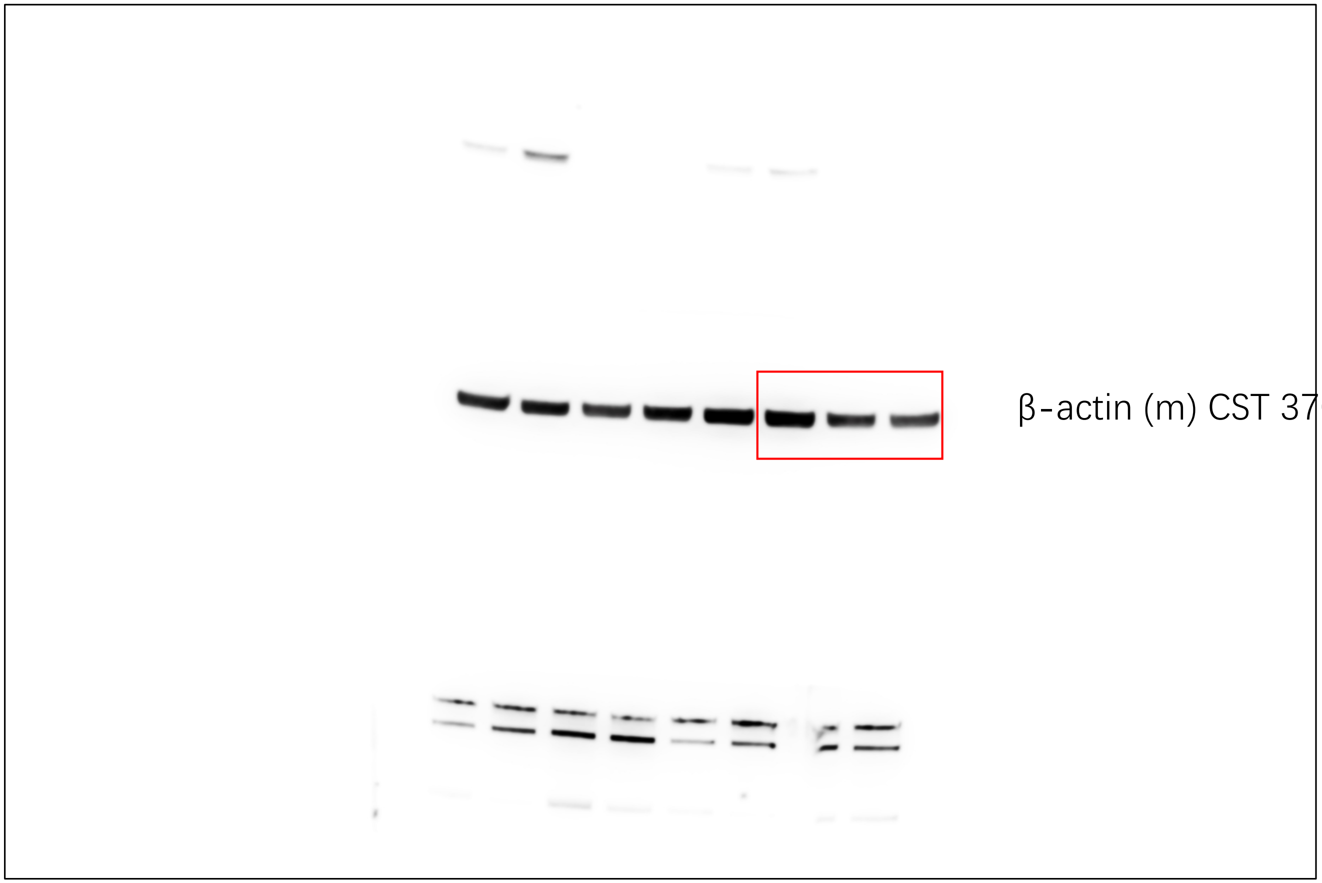

β-actin (m) CST 3700s

Full unedited blots for Figure 3C

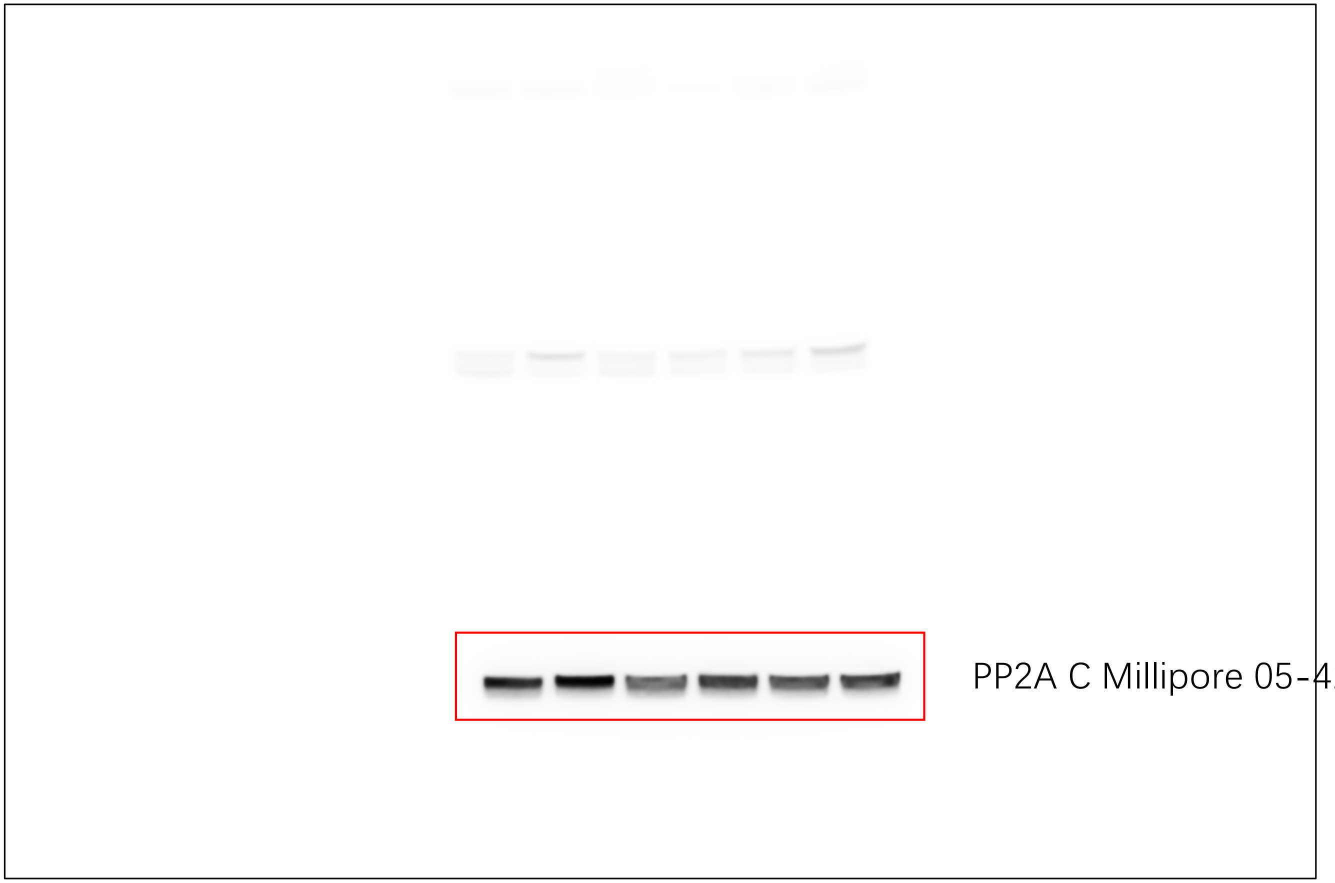

PP2A C Millipore 05-421

Full unedited blots for Figure 3C

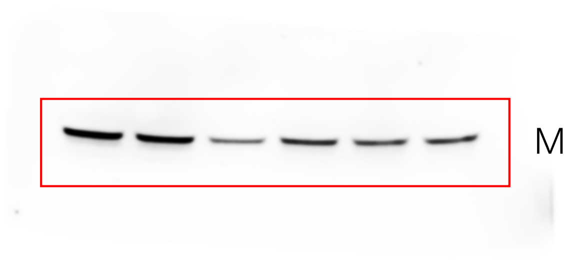

Full unedited blots for Figure 3C

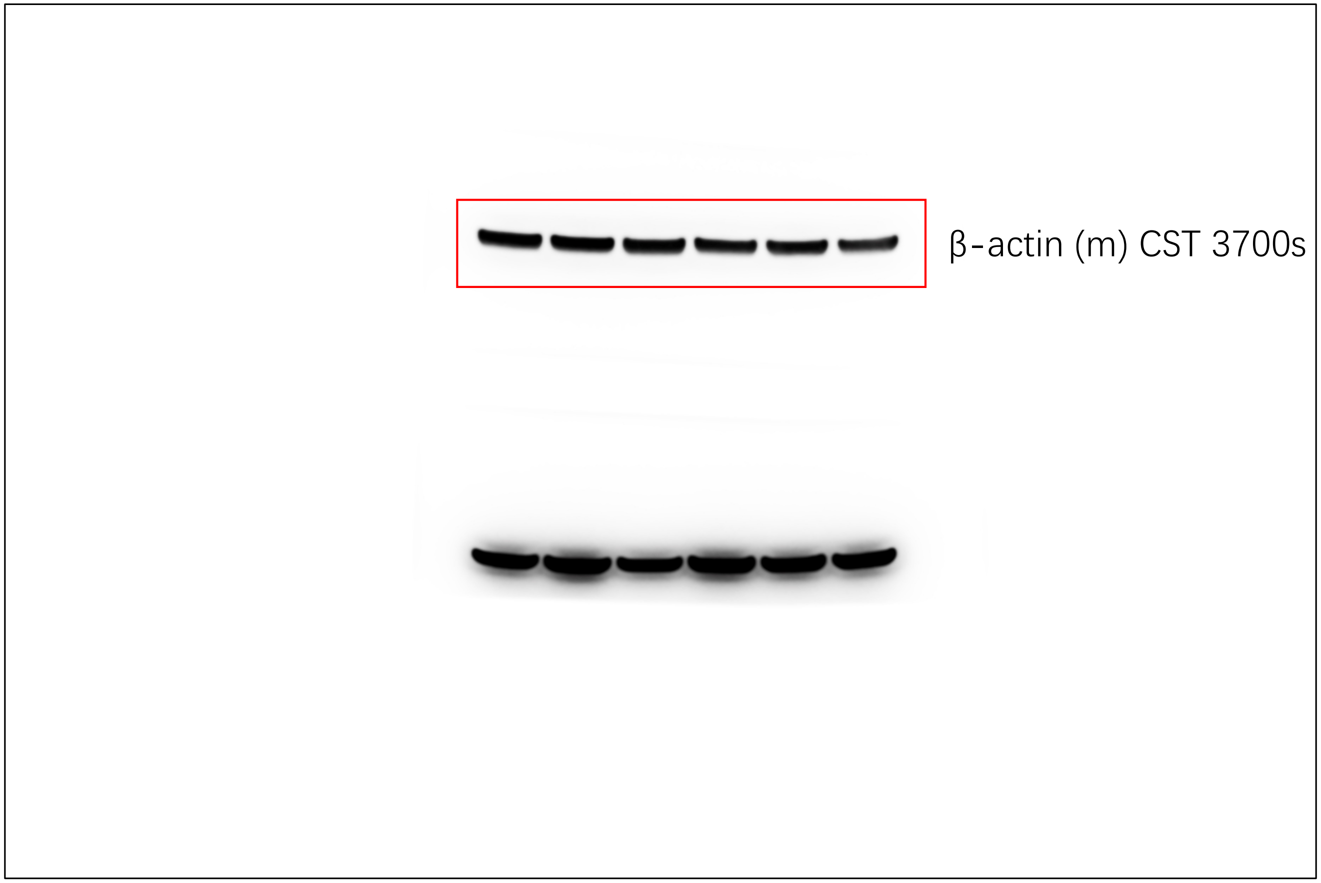

Full unedited blots for Figure 3D

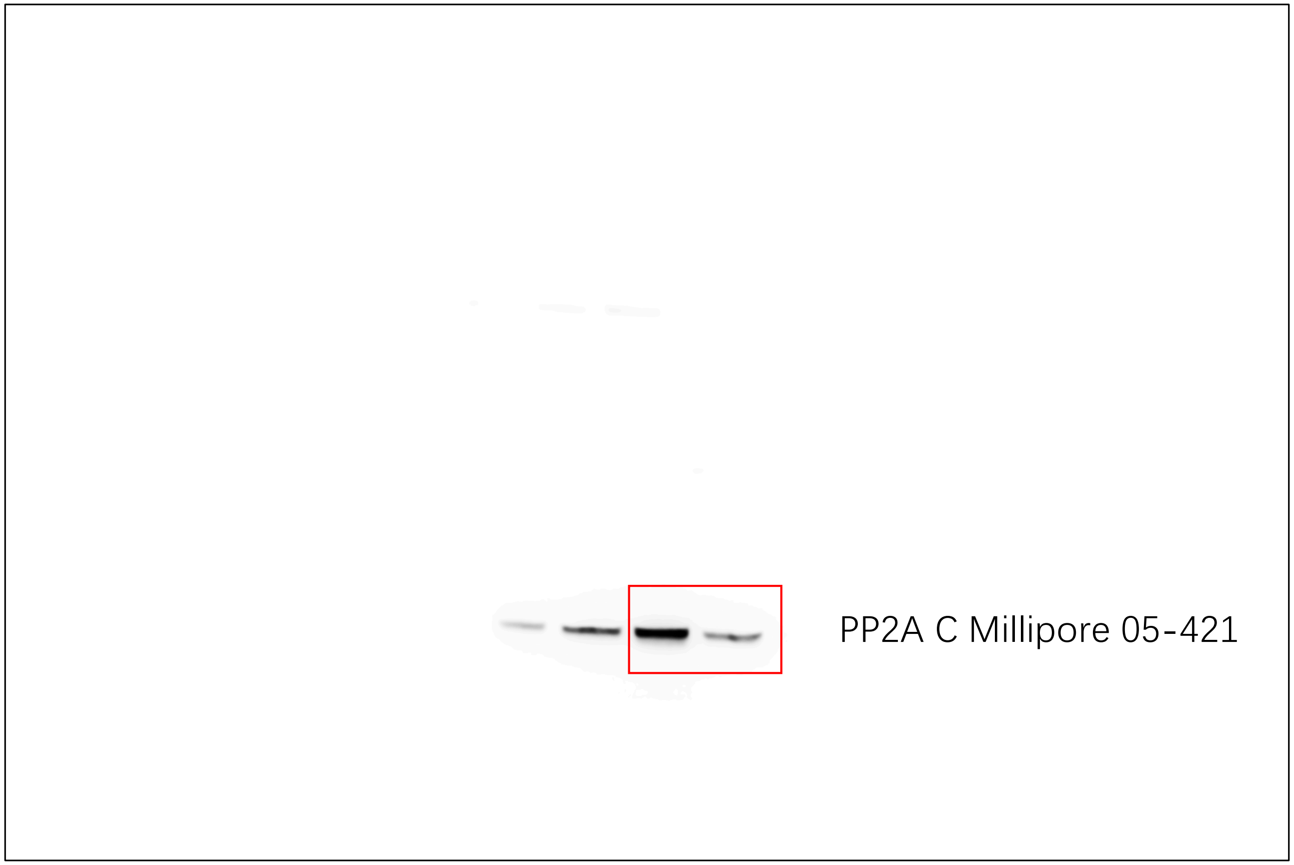

Full unedited blots for Figure 3D

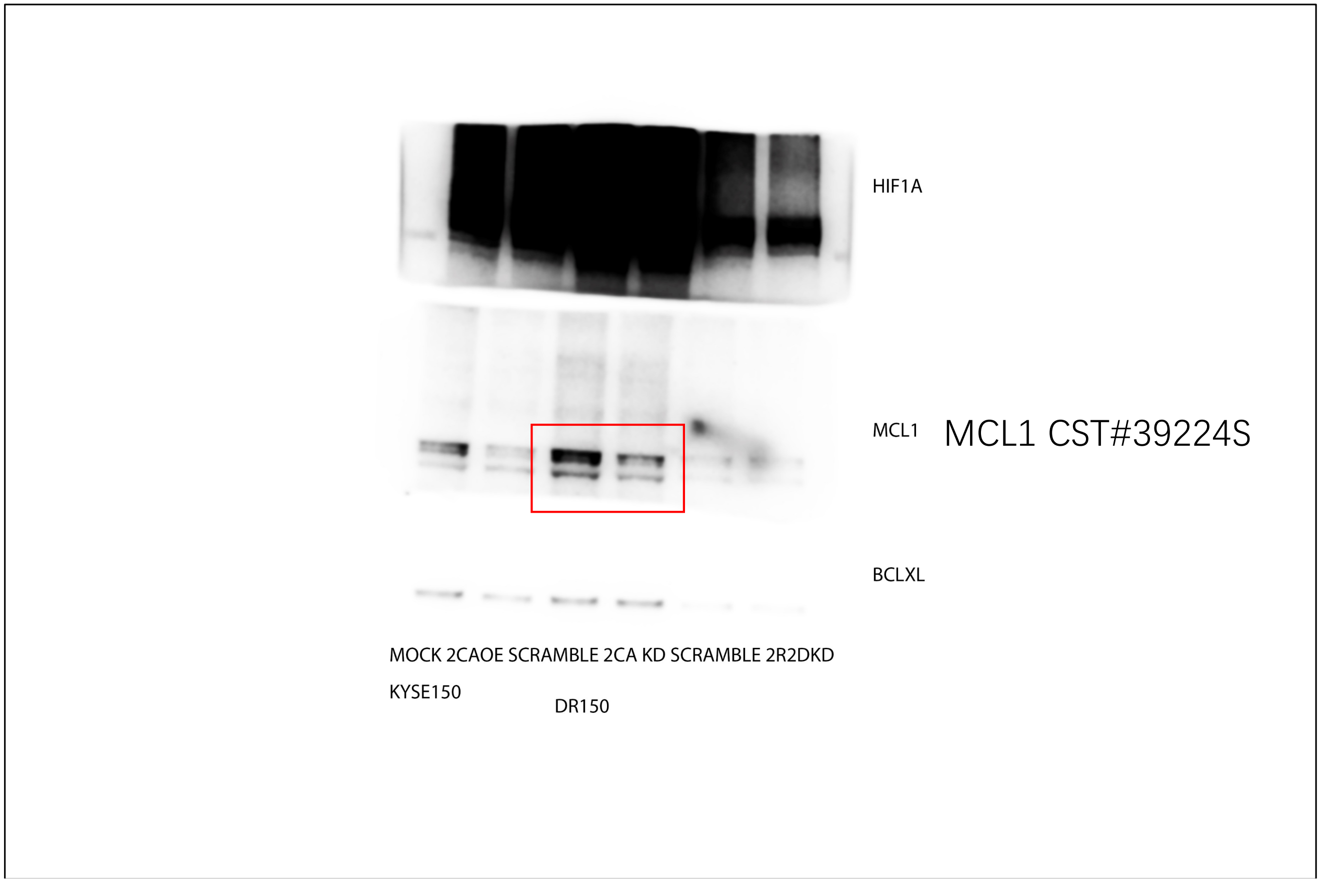

Full unedited blots for Figure 3D

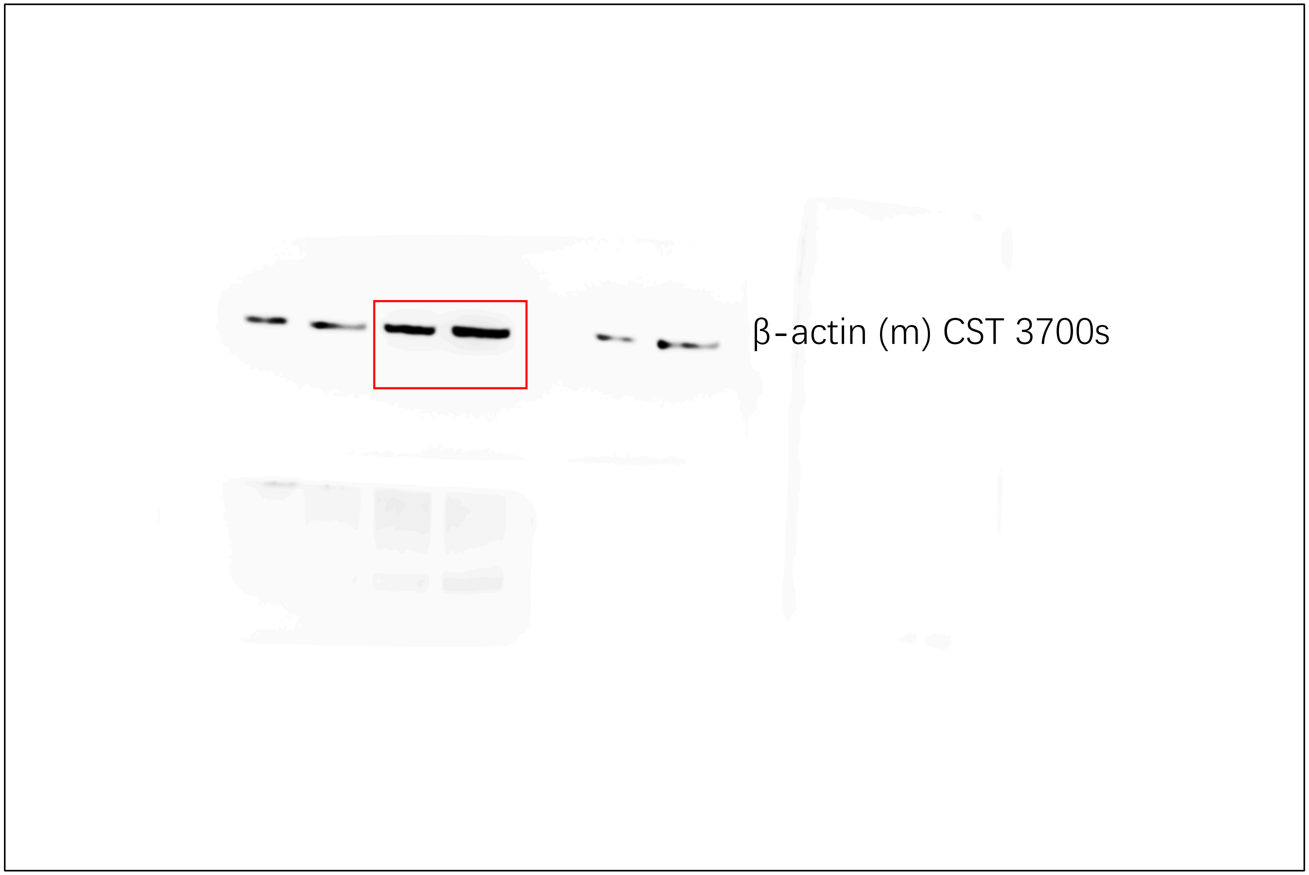

Full unedited blots for Figure 4B

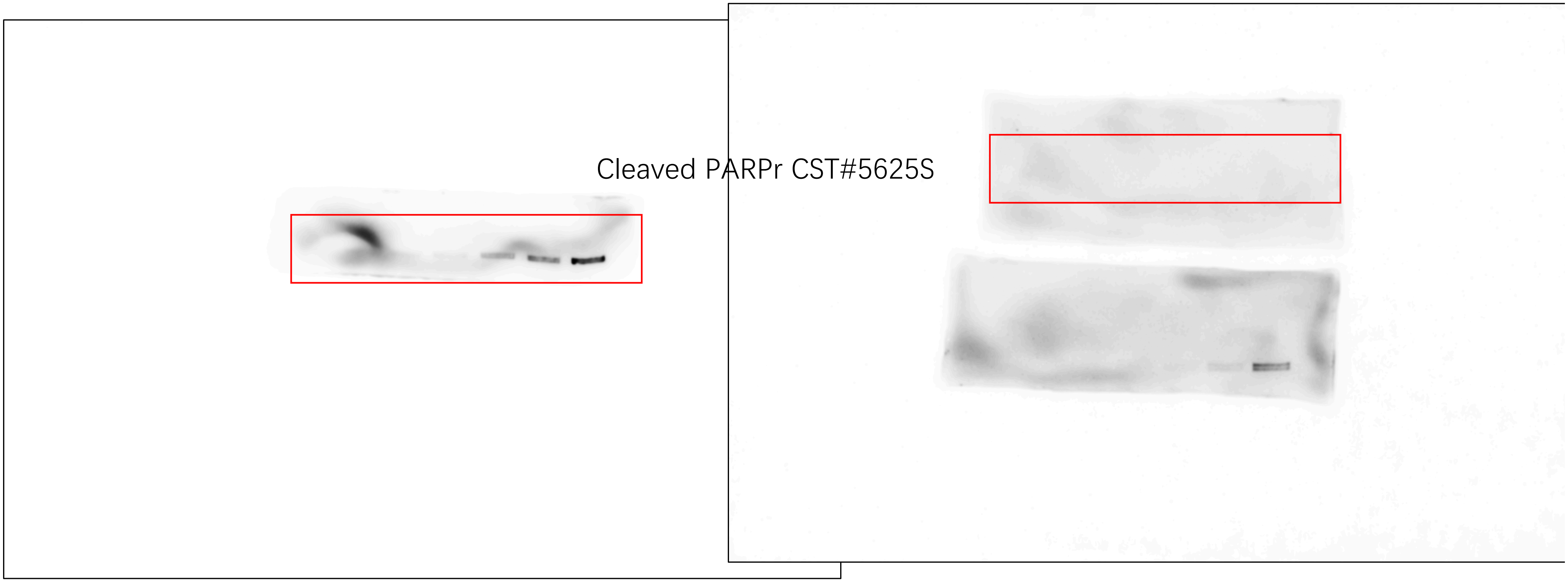

Full unedited blots for Figure 4B

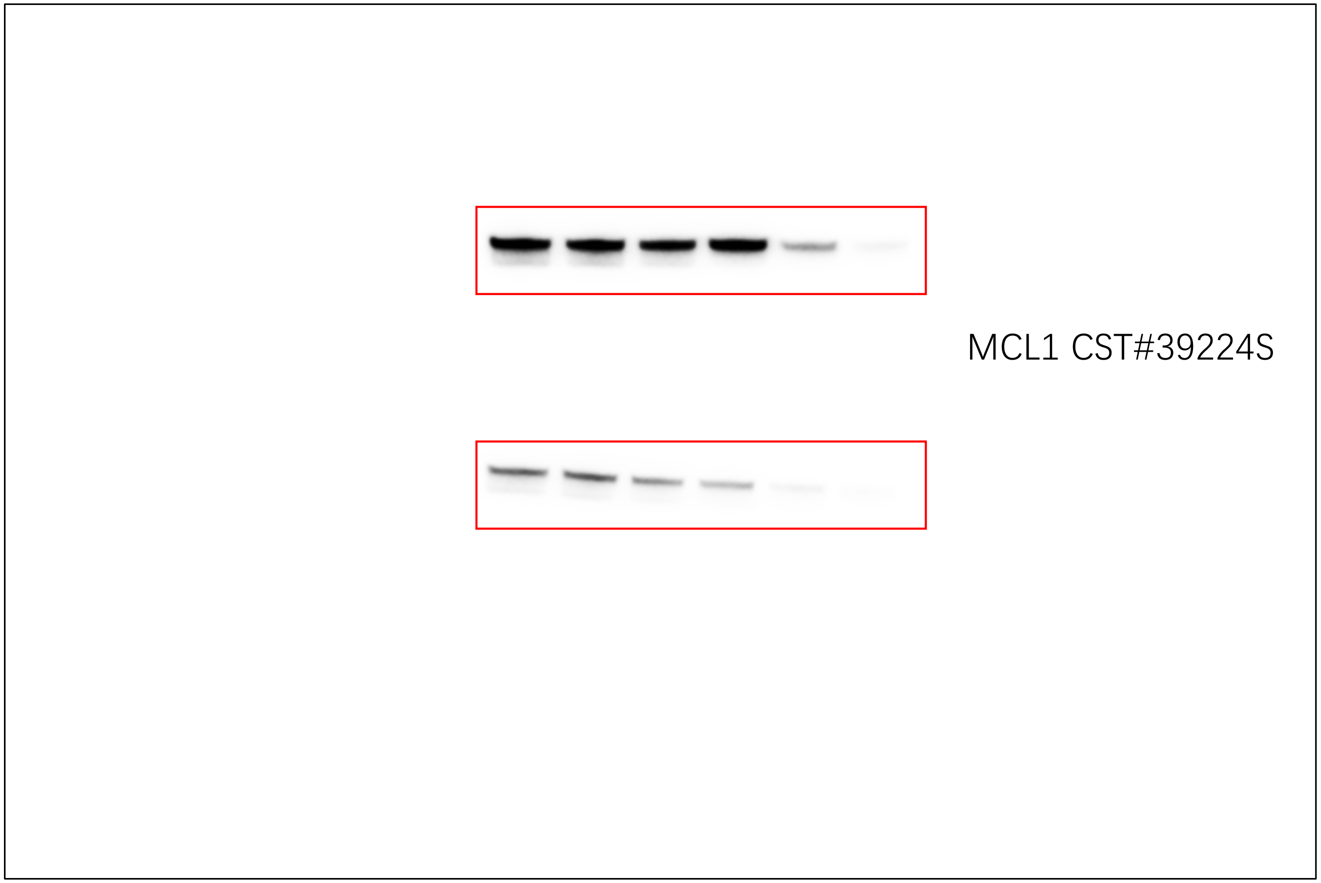

Full unedited blots for Figure 4B

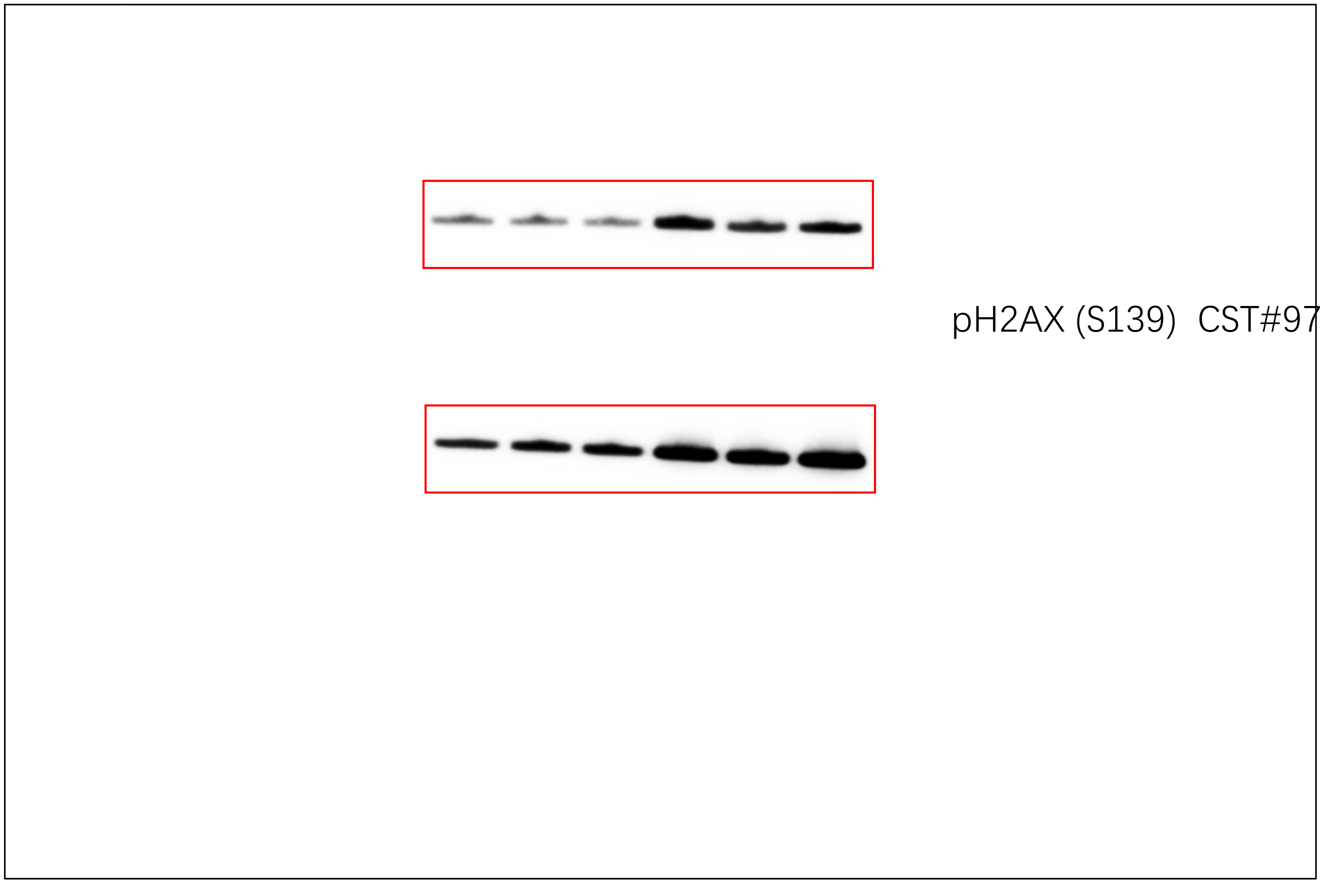

pH2AX (S139) CST#9718S

Full unedited blots for Figure 4B

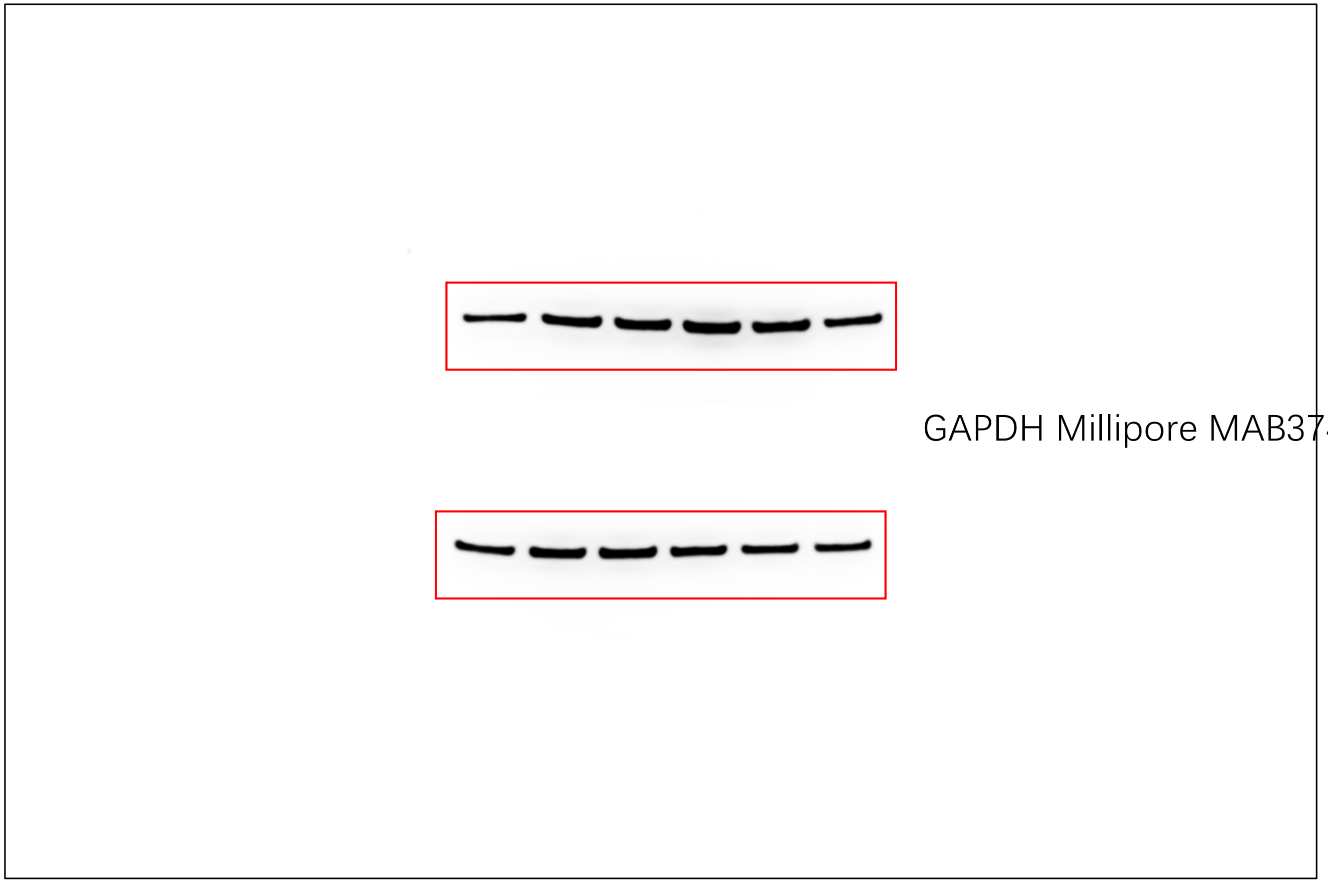

GAPDH Millipore MAB374

Full unedited blots for Figure 4C

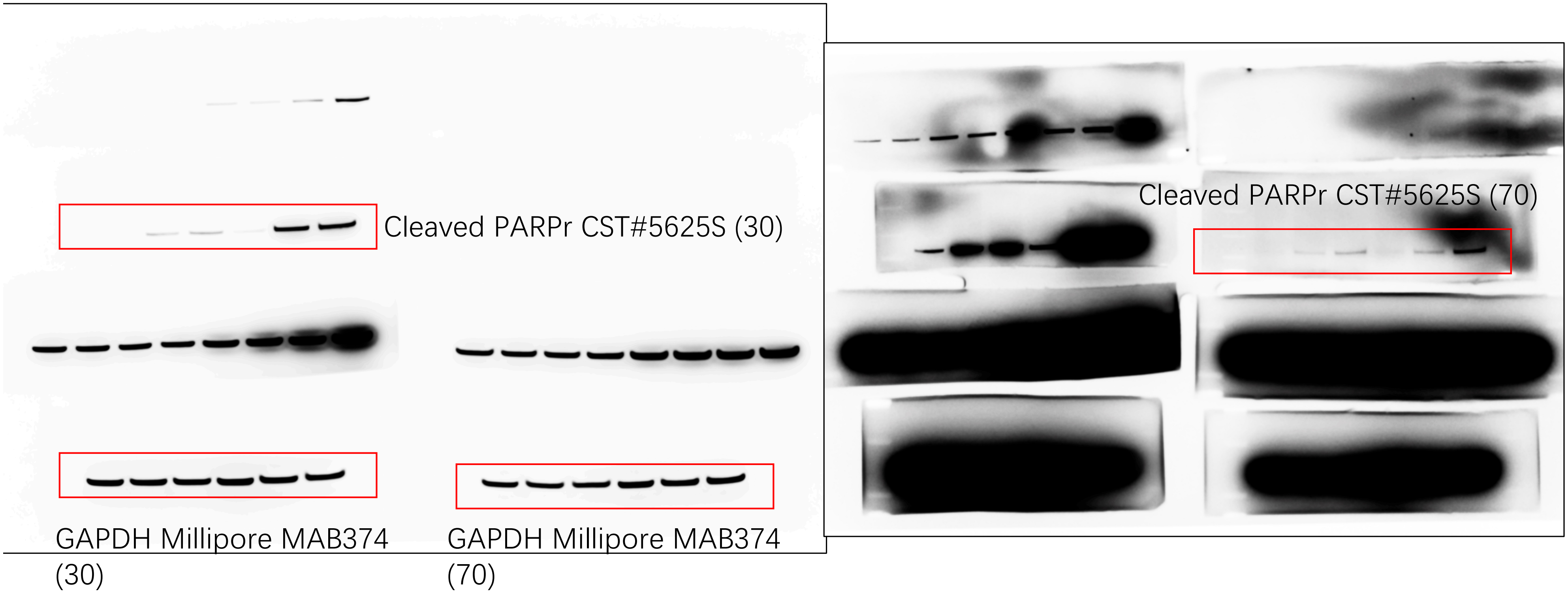

Full unedited blots for Figure 4C

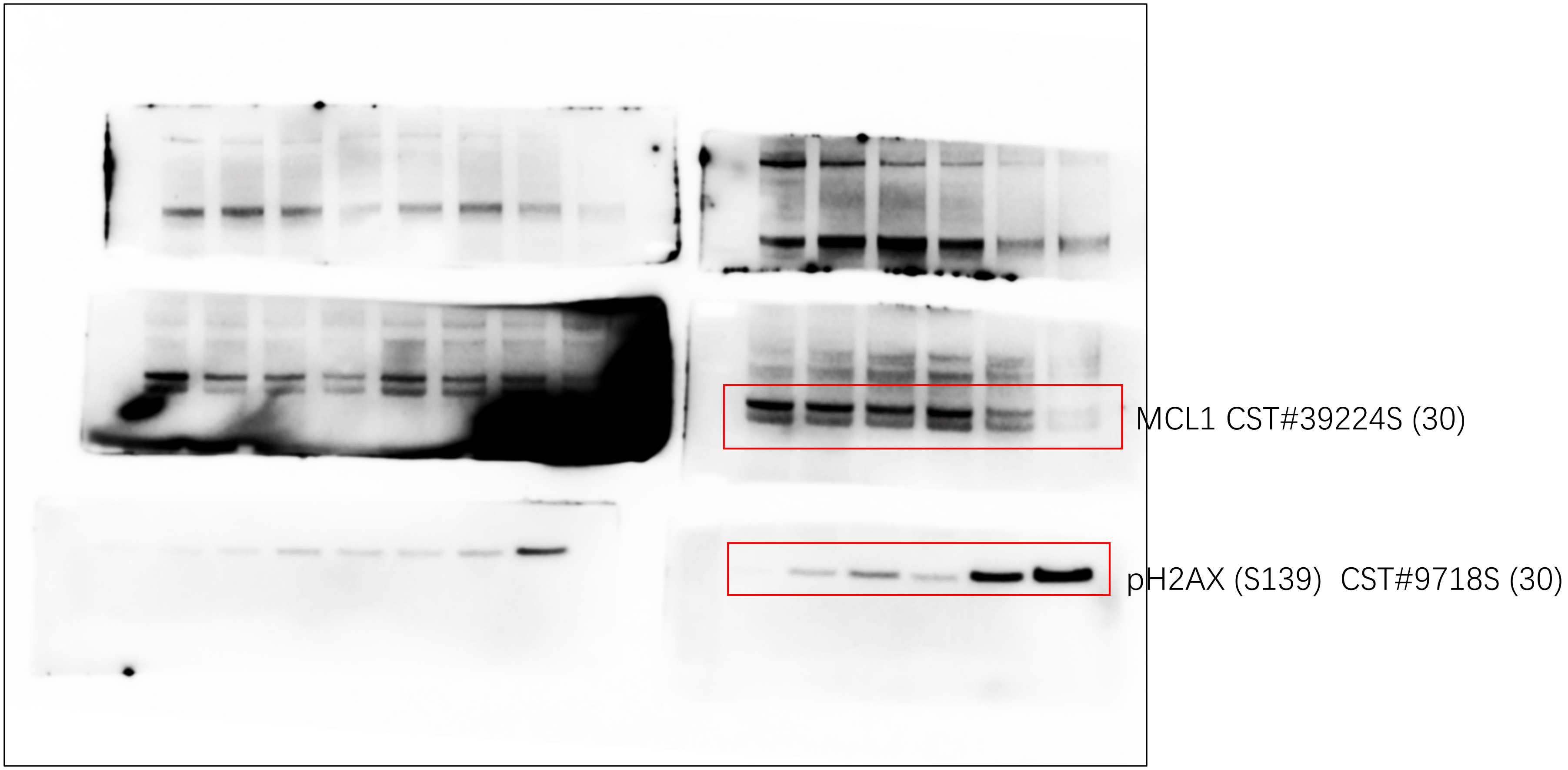

Full unedited blots for Figure 4C

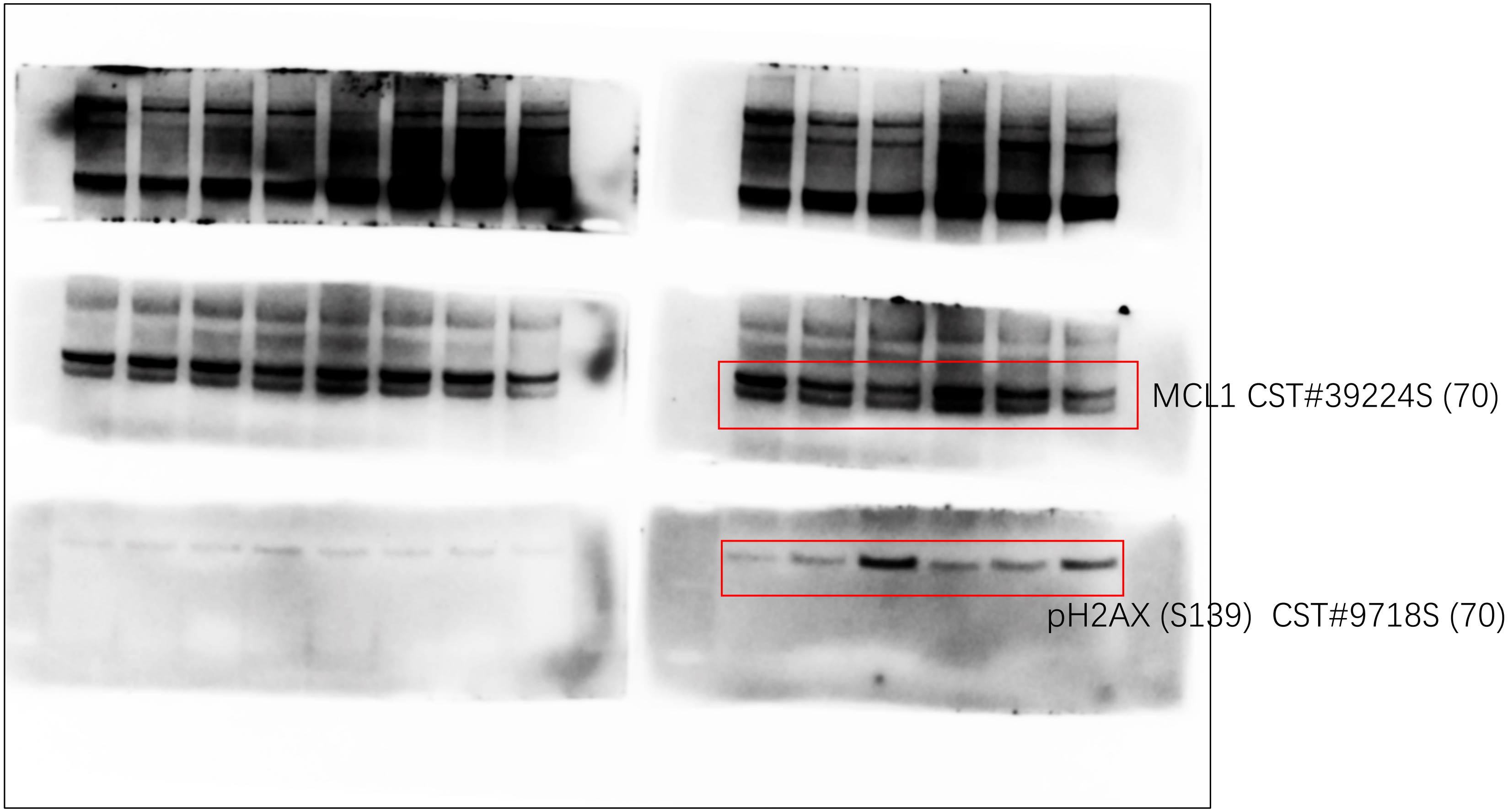

Supplement: Supplementary file 1 [file cancers-13-04766-s001.zip › cancers-1355481-original-images.pdf]
