# Supplementary material for: Pharmacological Inhibition of PP2A Overcomes Nab-Paclitaxel Resistance by Downregulating MCL1 in Esophageal Squamous Cell Carcinoma (ESCC)
Source: Cancers (Basel). 2021 Sep 23;13(19):4766. doi: 10.3390/cancers13194766 (PMC8507542; doi:10.3390/cancers13194766)
Supplement: Supplementary file 1 [file cancers-13-04766-s001.zip › cancers-1355481-supplementary.pdf]

Supplementary figures

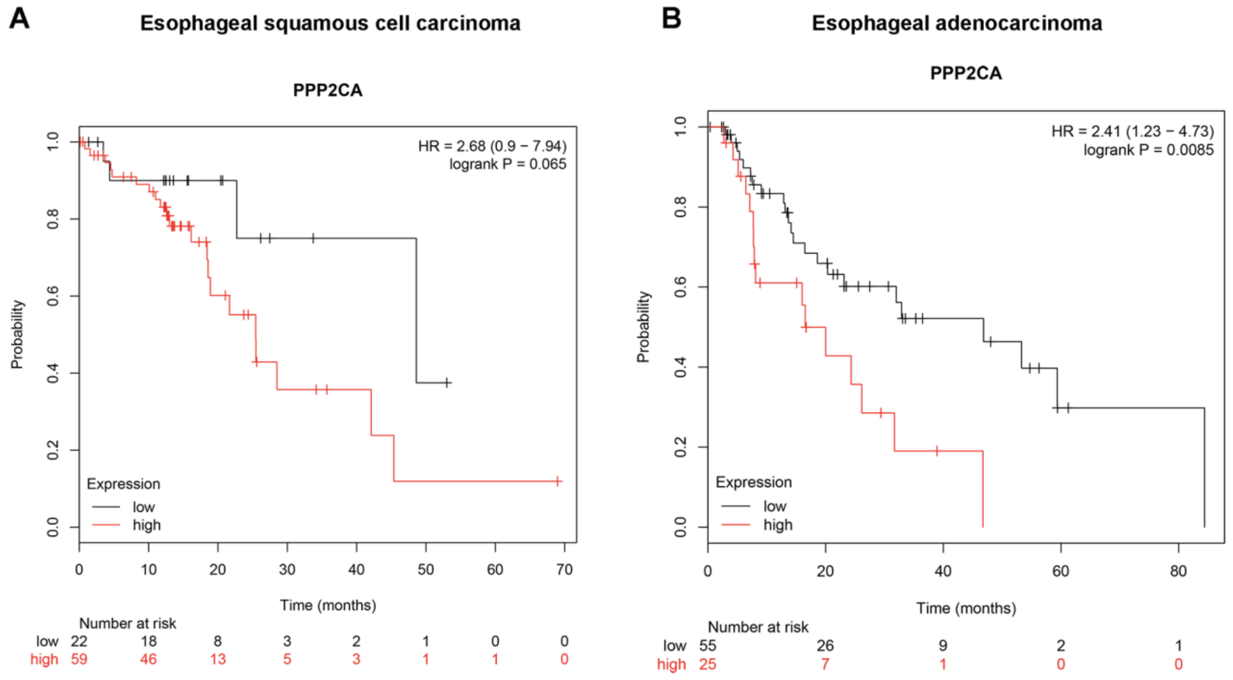

Figure S1. PPP2CA expression is negatively correlated with overall survival of esophageal cancer. (A-B) High PPP2CA expression leads to shorter overall survival in both esophageal squamous cell carcinoma (ESCC) (A, P=0.065, log-rank test) and esophageal adenocarcinoma (EAC) patients (B, P=0.0085, log-rank test). The analysis was based on the Kaplan-Meier Plotter datasets: in ESCC, n (high PPP2CA expression) = 59 and n (low PPP2CA expression) = 22; in EAC, n (high PPP2CA expression) = 25 and n (low PPP2CA expression) = 55.

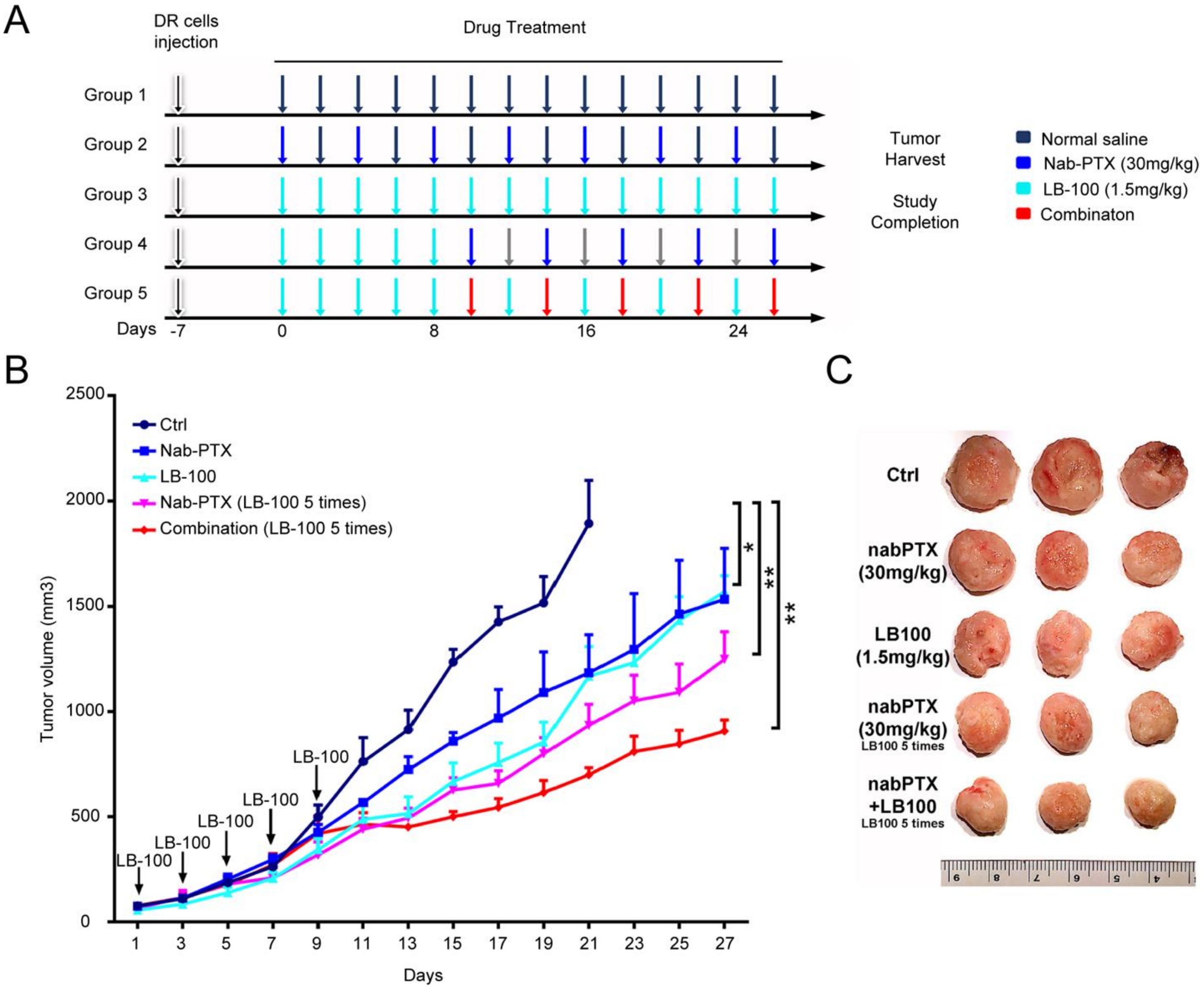

Figure S2. LB-100 treatment re-sensitized DR70 xenograft models to nab-PTX. (A) Experimental design using DR70 xenograft mouse models. Group 1: Normal saline i.p. every other day; Group 2: nab-PTX (30 mg/kg), i.v. every four days; Group 3: LB-100 (1.5 mg/kg) i.p. every other day; Group 4: LB-100 treatment for 5 doses and then switched to nab-PTX treatment; Group 5: LB-100 treatment for 5 doses and then switched to combined treatment. (B) DR70 xenograft growth curve of each group. Data are shown as mean ± SEM. P values were calculated using two-tailed Student's t test (*P<0.05, **P<0.01). (C) Representative tumor pictures of each group.
